# Supplementary figures and images for: A novel esterase regulates Klebsiella pneumoniae hypermucoviscosity and virulence
Source: PLoS Pathog. 2024 Oct 31;20(10):e1012675. doi: 10.1371/journal.ppat.1012675 (PMC11556721; doi:10.1371/journal.ppat.1012675)

S1 Fig. Top 10 genes enriched from differentially expressed genes.

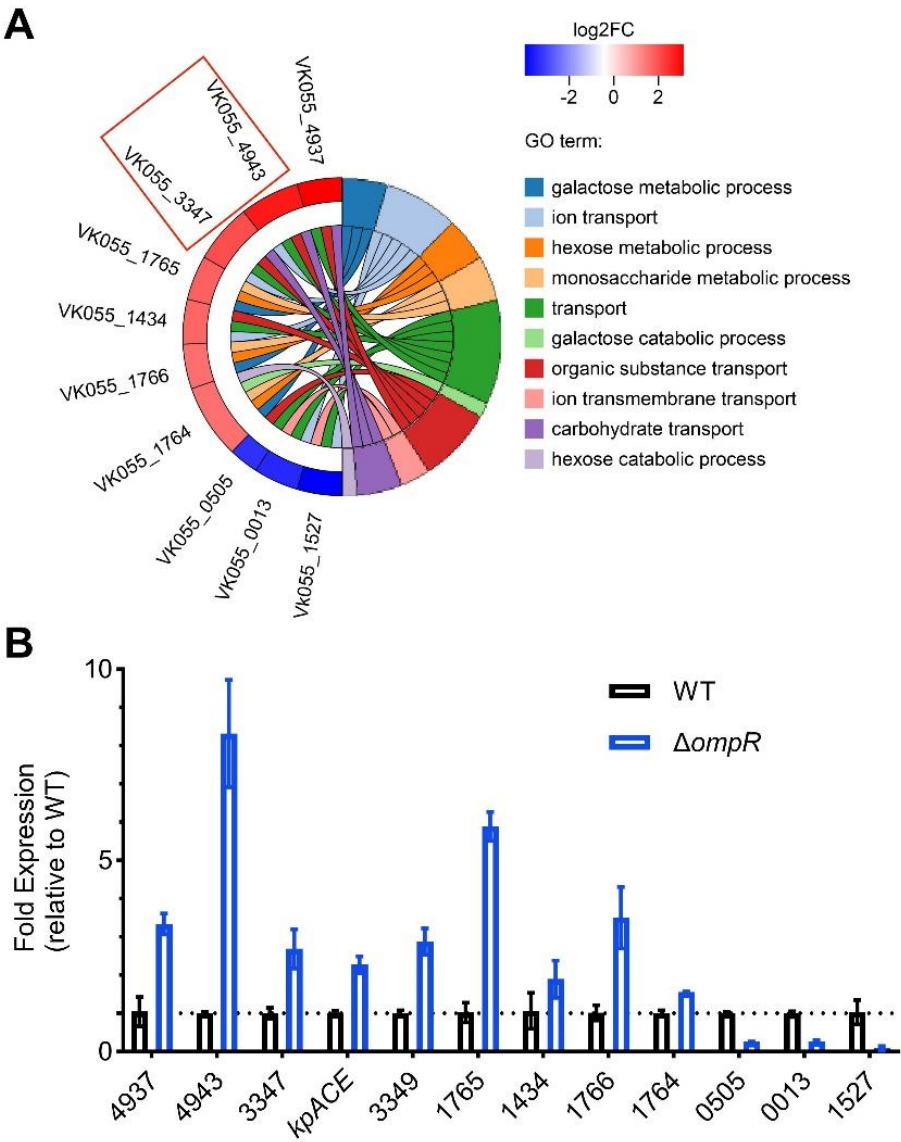

Supplement: S1 Fig — GO enrichment analysis of differentially expressed genes between the wild-type and ΔompR mutant cells at the log growth phase. (A) Chord diagram of top 10 GO terms. The top 10 genes are ranked by log2FC (Fold change) on the left side of the circle, and the correlating GO terms are on the right side. The values of log2FC were presented in S1 Table. The genes annotated as carbohydrate porin are highlighted with a red rectangle. (B) The transcriptional level of select genes. The transcriptional expression of select genes was quantified using the qRT-PCR assay. The expression level of each gene in the ΔompR mutant were normalized to that of wild-type (WT). The dotted line denotes the mean value of wild-type. (PDF) [file ppat.1012675.s001.pdf]

**S2 Fig. Binding of recombinant OmpR to the promoter region of VK055\_3347.**

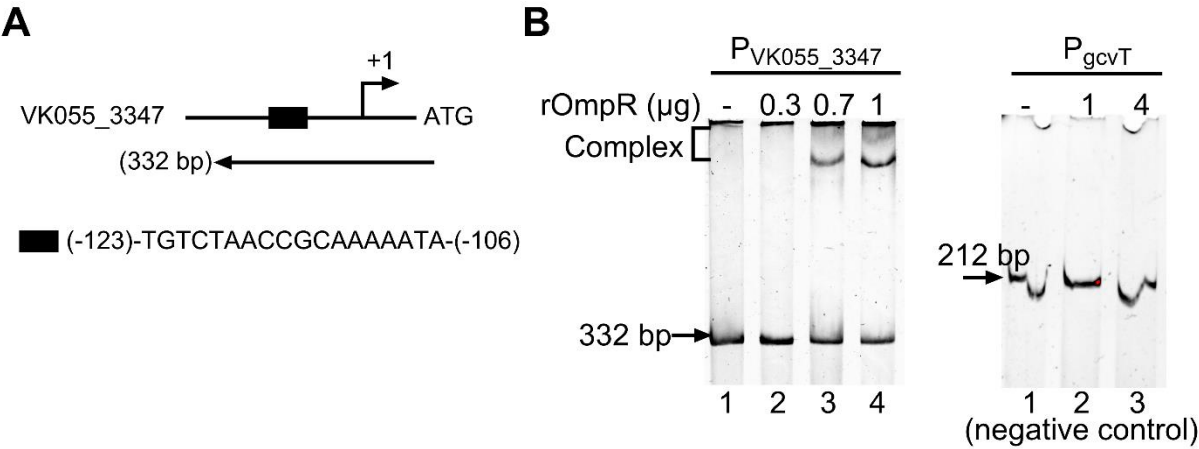

Supplement: S2 Fig — (A) Diagrams of the promoter region of the VK055_3347 locus (not drawn to scale). The black box represents a putative OmpR binding sequence. Nucleotide numberings are relative to the VK055_3347 ATG codon, respectively. The relative position and length of the DNA fragments used in EMSA are shown. (B) EMSA results of rOmpR. The PVK055_3347 DNA fragment was mixed with increasing concentrations of phosphorylated rOmpR at 0, 0.3, 0.7, and 1 μg (Lanes 1–4). The PgcvT DNA fragment was used as a negative control, mixing with phosphorylated rOmpR at 0, 1 and 4 μg (Lanes 1–3). DNA bands were detected by Gelred staining. The positions of DNA fragments that had not shifted were labeled. (PDF) [file ppat.1012675.s002.pdf]

**S6 Fig. Kinetic activity of recombinant KpACE.**

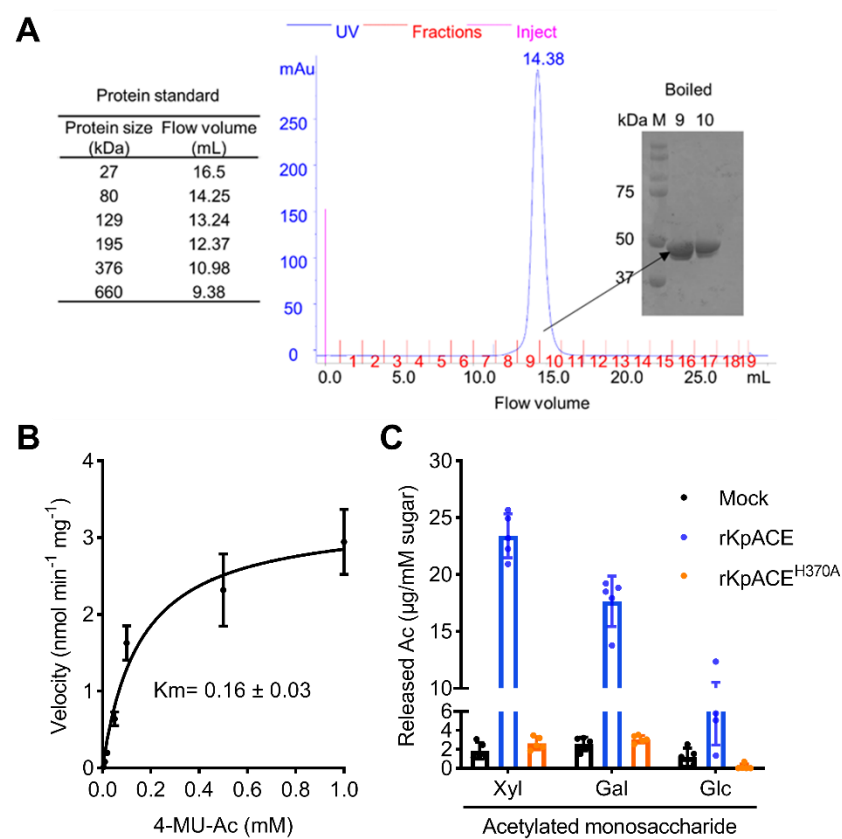

Supplement: S6 Fig — (A) Size-exclusion chromatography purification of recombinant KpACE. Purification of the rKpACE protein using molecular size Superdex 200. Samples corresponding to the peak position at 14.38 mL (75.6 kDa) were collected, and the presence of the KpACE protein was confirmed by 12% SDS-PAGE gel electrophoresis followed by protein staining. (B) Kinetic acetylesterase activity of rKpACE determined with 4-MU-Ac at pH 7.4. The data were nonlinearly fitted to the Michaelis-Menten equation by GraphPad Prism v9.3.1. (C) Hydrolytic activities of KpACE on three fully acetylated monosaccharides. Acetylated monosaccharides include 1,2,3,4-Tetra-O-acetyl-β-D-xylopyranose (Xyl), 1,2,3,4,6-Penta-O-acetyl-β-D-galactopyranose (Gal), 1,2,3,4,6-β-D-glucose pentaacetate (Glu). The activity was determined by measuring the amounts of acetic acids released from the substrates by rKpACE and its variants. Data are from six biological replicates. The unpaired t-test was performed to determine significant differences. (PDF) [file ppat.1012675.s006.pdf]

**S7 Fig. Extracellular localization of the KpACE protein.**

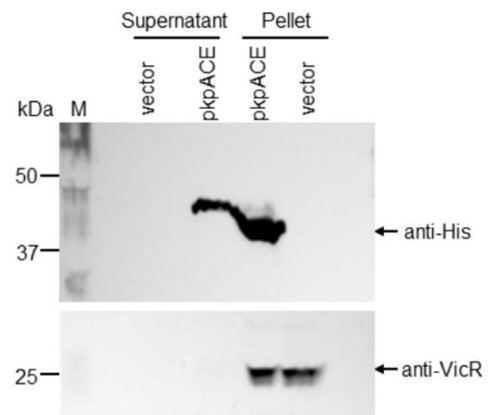

Supplement: S7 Fig — Immunoblotting of culture supernatants and cell pellets was performed on K. pneumoniae cells carrying pkpACE (TH16356), which were cultured in an LB medium to OD600nm of 1.0. All samples were boiled for 10 minutes, and 15 μL of each sample was loaded onto a 12% SDS-PAGE gel. The KpACE-6×His fusion protein expressed by TH16356 was probed using a 6×His Tag monoclonal antibody (Invitrogen). The response regulator VicR, a known intracellular protein, was employed as a negative control to verify the localization of KpACE. Comparing the immunoblotting results of VicR and the KpACE-6×His fusion protein, we conclude that any detected signal for KpACE in the supernatant was not due to cell lysis or contamination with intracellular proteins. (PDF) [file ppat.1012675.s007.pdf]

**S8 Fig. The monosaccharide compositions of EPS.**

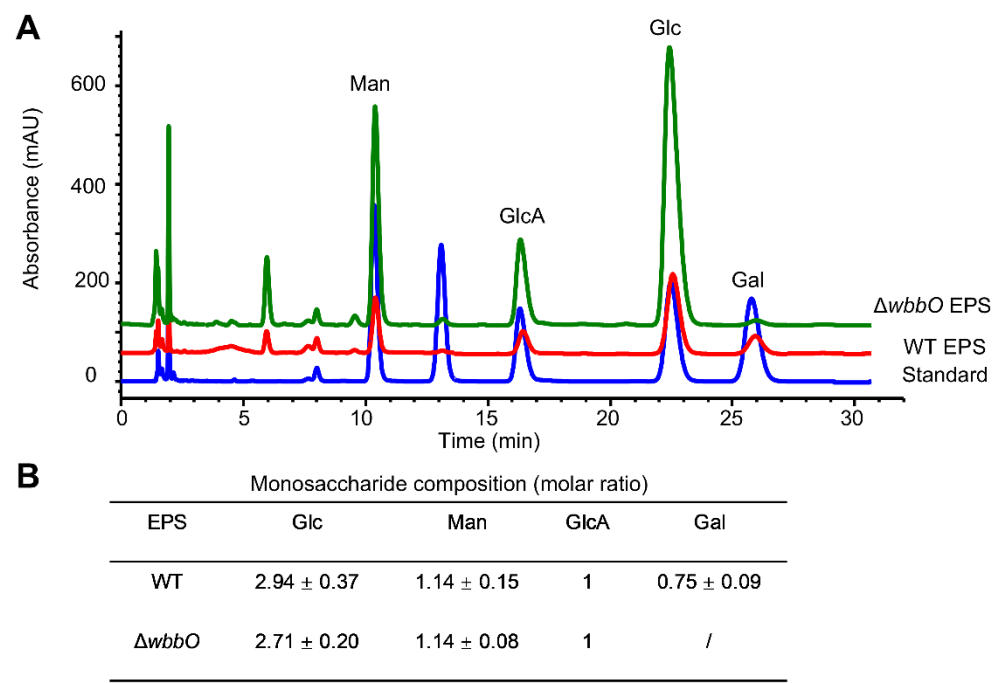

Supplement: S8 Fig — (A) Monosaccharide compositions of wild-type EPS determined using High-Performance Liquid Chromatography (HPLC). (B) The molar ratio of monosaccharides in EPS from wild-type and ΔwbbO strains. (PDF) [file ppat.1012675.s008.pdf]
