# Supplementary material for: A novel esterase regulates Klebsiella pneumoniae hypermucoviscosity and virulence
Source: PLoS Pathog. 2024 Oct 31;20(10):e1012675. doi: 10.1371/journal.ppat.1012675 (PMC11556721; doi:10.1371/journal.ppat.1012675)

S3 Fig. Effects of KpACE deficiency on virulence in a pneumonia model using both female and male mice.

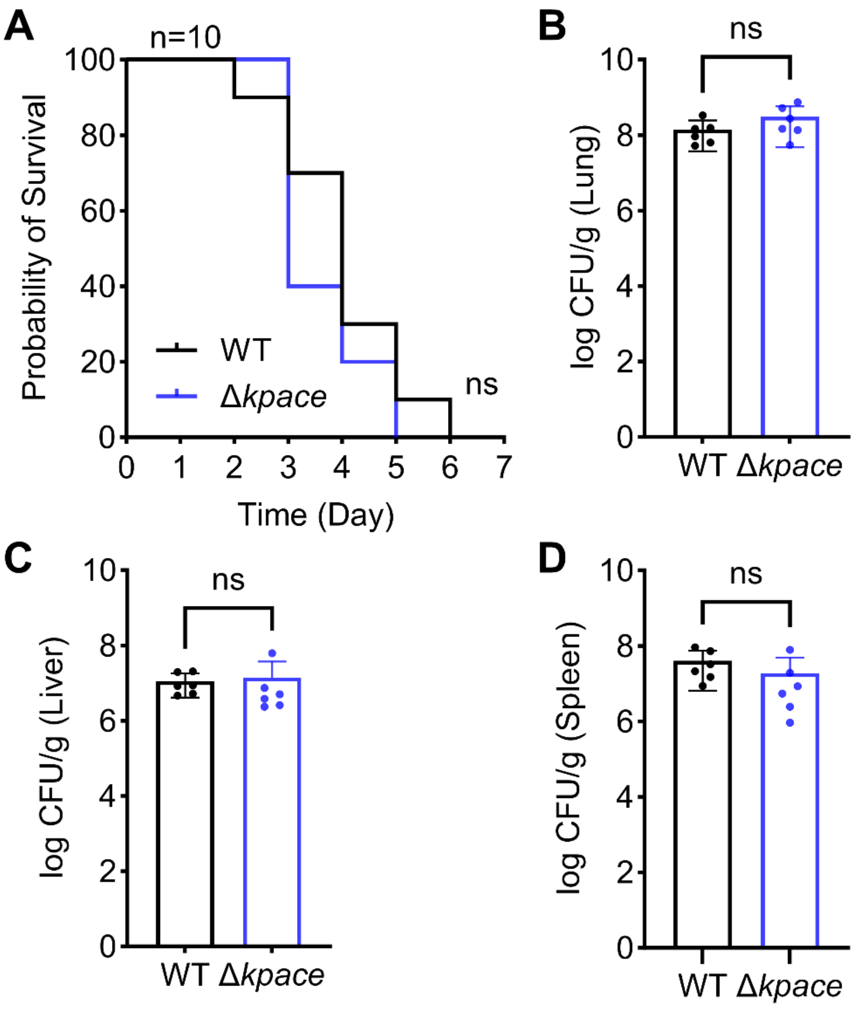

Supplement: S3 Fig — (A) Survival rates of mice (6 female and 4 male) infected by K. pneumoniae variants. Mouse survival rates were monitored 7 days post intranasal infection with 2,000 CFU of various ATCC43816 strains. The Gehan-Breslow-Wilcoxon test was performed to compare the survival rates. (B-D) Bacterial colonization in different organs of infected mice (3 female and 3 male). Bacterial burdens in lungs (B), livers (C), and spleens (D) were determined 48 h post intranasal inoculation. Each dot represents one mouse. An unpaired t-test was performed to determine the statistical significance. (PDF) [file ppat.1012675.s003.pdf]
