# Supplementary material for: A novel esterase regulates Klebsiella pneumoniae hypermucoviscosity and virulence
Source: PLoS Pathog. 2024 Oct 31;20(10):e1012675. doi: 10.1371/journal.ppat.1012675 (PMC11556721; doi:10.1371/journal.ppat.1012675)
Supplement: S4 Fig — (A) The distribution of KL-serotypes of 1503 K. pneumoniae strains harboring KpACE homologues. 1503 KpACE homologues were retrieved from the NCBI database as detailed in the Materials and Methods section. The K-serotype of each strain was determined using the Kleborate tool, which performed K-locus typing based on their genomic sequences. KL-types with a count of less than 30, combined together as ’others’, were not included. (B) The multisequence alignment of KpACE homologues from representative K. pneumoniae K-serotypes. The alignment was created and visualized using DNAMAN 9.0 software. The identity of the ten KpACE homologues is 99.85%. Amino acid residues that are identical across sequences are represented in black text on a white background, while variable residues are depicted as black text on a pink background. (PDF) [file ppat.1012675.s004.pdf]

S4 Fig. Multisequence alignment of KpACE homologues in different *K. pneumoniae* serotypes.

A

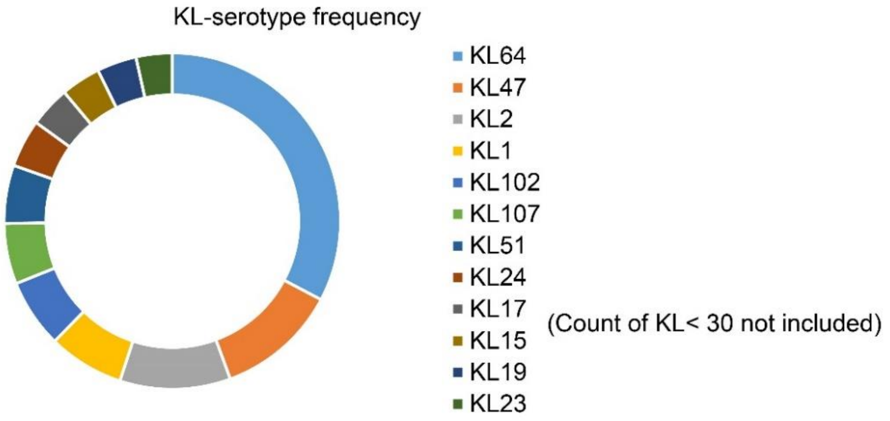

B

```
ATCC43816__K2_        MKRHAIYFALALAGAAFTLGAAPLPAMPDPSLPVSHFITQVNADKSITYRLFAPCARRVSIVTGATPDSFVSHDMTKAAD   80
Kp52.145__K2_         MKRHAIYFALALAGAAFTLGAAPLPAMPDPSLPVSHFITQVNADKSITYRLFAPCARRVSIVTGATPDSFVSHDMTKAAD   80
KpCG43__K2_           MKRHAIYFALALAGAAFTLGAAPLPAMPDPSLPVSHFITQVNADKSITYRLFAPCARRVSIVTGATPDSFVSHDMTKAAD   80
NTUH-K2044__K1_       MKRHAIYFALALAGAAFTLGAAPLPAMPDPSLPVSHFITQVNADKSITYRLFAPCARRVSIVTGATPDSFVSHDMTKAAD   80
KpSGH10__K1_          MKRHAIYFALALAGAAFTLGAAPLPAMPDPSLPVSHFITQVNADKSITYRLFAPCARRVSIVTGATPDSFVSHDMTKAAD   80
TH12896__K23_         MKRHAIYFALALAGAAFTLGAAPLPAMPDPSLPVSHFITQVNADKSITYRLFAPCARRVSIVTGATPDSFVSHDMTKAAD   80
KpMGH78578__K50_      MKRHAIYFALALAGAAFTLGAAPLPAMPDPSLPVSHFITQVNADKSITYRLFAPCARRVSIVTGATPDSFVSHDMTKAAD   80
KPNIH29__KL64_        MKRHAIYFALALAGAAFTLGAAPLPAMPDPSLPVSHFITQVNADKSITYRLFAPCARRVSIVTGATPDSFVSHDMTKAAD   80
ATCC35657__KL64_      MKRHAIYFALALAGAAFTLGAAPLPAMPDPSLPVSHFITQVNADKSITYRLFAPCARRVSIVTGATPDSFVSHDMTKAAD   80
NZ_CP098770.1__K47_   MKRHAIYFALALAGAAFTLGAAPLPAMPDPSLPVSHFITQVNADKSITYRLFAPCARRVSIVTGATPDSFVSHDMTKAAD   80

ATCC43816__K2_        GVVTVKSEPMKPNLYEYYFDVDGFRSICTGSRYQKPQRQVNTSLILVPGSILDDREVAHGDLRTLTYHSKALNAERRLYV   160
Kp52.145__K2_         GVVTVKSEPMKPNLYEYYFDVDGFRSVCTGSRYQKPQRQVNTSLILVPGSILDDREVAHGDLRTLTYHSKALNAERRLYV   160
KpCG43__K2_           GVVTVKSEPMKPNLYEYYFDVDGFRSVCTGSRYQKPQRQVNTSLILVPGSILDDREVAHGDLRTLTYHSKALNAERRLYV   160
NTUH-K2044__K1_       GVVTVKSEPMKPNLYEYYFDVDGFRSVCTGSRYQKPQRQVNTSLILVPGSILDDREVAHGDLRTLTYHSKALNAERRLYV   160
KpSGH10__K1_          GVVTVKSEPMKPNLYEYYFDVDGFRSVCTGSRYQKPQRQVNTSLILVPGSILDDREVAHGDLRTLTYHSKALNAERRLYV   160
TH12896__K23_         GVVTVKSEPMKPNLYEYYFDVDGFRSVCTGSRYQKPQRQVNTSLILVPGSILDDREVAHGDLRTLTYHSKALNAERRLYV   160
KpMGH78578__K50_      GVVTVKSEPMKPNLYEYYFDVDGFRSVCTGSRYQKPQRQVNTSLILVPGSILDDREVAHGDLRTLTYHSKALNAERRLYV   160
KPNIH29__KL64_        GVVTVKSEPMKPNLYEYYFDVDGFRSVCTGSRYQKPQRQVNTSLILVPGSILDDREVAHGDLRTLTYHSKALNAERRLYV   160
ATCC35657__KL64_      GVVTVKSEPMKPNLYEYYFDVDGFRSVCTGSRYQKPQRQVNTSLILVPGSILDDREVAHGDLRTLTYHSKALNAERRLYV   160
NZ_CP098770.1__K47_   GVVTVKSEPMKPNLYEYYFDVDGFRSVCTGSRYQKPQRQVNTSLILVPGSILDDREVAHGDLRTLTYHSKALNAERRLYV   160

ATCC43816__K2_        VTPPGYSCTGDPLPVLYFYHGFGDSGLSAIDQGRIPQIMDNLLAEGKIKPMLVVVPCTETDIPEAVAENFPPQERRKTFY   240
Kp52.145__K2_         VTPPGYSCTGDPLPVLYFYHGFGDSGLSAIDQGRIPQIMDNLLAEGKIKPMLVVVPCTETDIPEAVAENFPPQERRKTFY   240
KpCG43__K2_           VTPPGYSCTGDPLPVLYFYHGFGDSGLSAIDQGRIPQIMDNLLAEGKIKPMLVVVPCTETDIPEAVAENFPPQERRKTFY   240
NTUH-K2044__K1_       VTPPGYSCTGDPLPVLYFYHGFGDSGLSAIDQGRIPQIMDNLLAEGKIKPMLVVVPCTETDIPEAVAENFPPQERRKTFY   240
KpSGH10__K1_          VTPPGYSCTGDPLPVLYFYHGFGDSGLSAIDQGRIPQIMDNLLAEGKIKPMLVVVPCTETDIPEAVAENFPPQERRKTFY   240
TH12896__K23_         VTPPGYRCTGDPLPVLYFYHGFGDSGLSAIDQGRIPQIMDNLLAEGKIKPMLVVVPCTETDIPEAVAEDFPPQERRKTFY   240
KpMGH78578__K50_      VTPPGYSCTGDPLPVLYFYHGFGDSGLSAIDQGRIPQIMDNLLAEGKIKPMLVVVPCTETDIPEAVAENFPPQERRKTFY   240
KPNIH29__KL64_        VTPPGYSCTGDPLPVLYFYHGFGDSGLSAIDQGRIPQIMDNLLAEGKIKPMLVVVPCTETDIPEAVAEDFPPQERRKTFY   240
ATCC35657__KL64_      VTPPGYSCTGDPLPVLYFYHGFGDSGLSAIDQGRIPQIMDNLLAEGKIKPMLVVVPCTETDIPEAVAENFPPQERRKTFY   240
NZ_CP098770.1__K47_   VTPPGYSCTGDPLPVLYFYHGFGDSGLSAIDQGRIPQIMDNLLAEGKIKPMLVVVPCTETDIPEAVAENFPPQERRKTFY   240

ATCC43816__K2_        PLNACAADKELMQDIIPLICARFNVRKCADGRALAGLSQGGYCALVSGMNHLESFGVLATFSGVTTTTVPNAGVEAQLKQ   320
Kp52.145__K2_         PLNACAADKELMQDIIPLICARFNVRKCADGRALAGLSQGGYCALVSGMNHLESFSVLATFSGVTTTTVPNAGVEAQLKQ   320
KpCG43__K2_           PLNACAADKELMQDIIPLICARFNVRKCADGRALAGLSQGGYCALVSGMNHLESFGVLATFSGVTTTTVPNAGVEAQLKQ   320
NTUH-K2044__K1_       PLNACAADKELMQDIIPLICARFNVRKCADGRALAGLSQGGYCALVSGMNHLESFGVLATFSGVTTTTVPNAGVEAQLKQ   320
KpSGH10__K1_          PLNACAADKELMQDIIPLICARFNVRKCADGRALAGLSQGGYCALVSGMNHLESFGVLATFSGVTTTTVPNAGVEAQLKQ   320
TH12896__K23_         PLNACAADKELMQDIIPLICARFNVRKCADGRALAGLSQGGYCALVSGMNHLESFGVLATFSGVTTTTVPNAGVEAQLKQ   320
KpMGH78578__K50_      PLNACAADKELMQDIIPLICARFNVRKCADGRALAGLSQGGYCALVSGMNHLESFGVLATFSGVTTTTVPNAGVEAQLKQ   320
KPNIH29__KL64_        PLNACAADKELMQDIIPLICARFNVRKCADGRALAGLSQGGYCALVSGMNHLESFGVLATFSGVTTTTVPNAGVEAQLKQ   320
ATCC35657__KL64_      PLNACAADKELMQDIIPLICARFNVRKCADGRALAGLSQGGYCALVSGMNHLESFGVLATFSGVTTTTVPNAGVEAQLKQ   320
NZ_CP098770.1__K47_   PLNACAADKELMQDIIPLICARFNVRKCADGRALAGLSQGGYCALVSGMNHLESFGVLATFSGVTTTTVPNAGVEAQLKQ   320

ATCC43816__K2_        PCAINKQLRNFTVVVGEKDSVTGKDIAGLKSELEKQQIKFDYHQYPGLNHEMDVVRPAYAEFVQKLFK   388
Kp52.145__K2_         PCAINKQLRNFTVVVGEKDSVTGKDIAGLKSELEKQQIKFDYHQYPGLNHEMDVVRPAYAEFVQKLFK   388
KpCG43__K2_           PCAINKQLRNFTVVVGEKDSVTGKDIAGLKSELEKQQIKFDYHQYPGLNHEMDVVRPAYAEFVQKLFK   388
NTUH-K2044__K1_       PCAINKQLRNFTVVVGEKDSVTGKDIAGLKSELEKQQIKFDYHQYPGLNHEMDVVRPAYAEFVQKLFK   388
KpSGH10__K1_          PCAINKQLRNFTVVVGEKDSVTGKDIAGLKSELEKQQIKFDYHQYPGLNHEMDVVRPAYAEFVQKLFK   388
TH12896__K23_         PCAINKQLRNFTVVVGEKDSVTGKDIAGLKSELEKQQIKFDYHQYPGLNHEMDVVRPAYAEFVQKLFK   388
KpMGH78578__K50_      PCAINKQLRNFTVVVGEKDSVTGKDIAGLKSELEKQQIKFDYYQYPGLNHEMDVVRPAYAEFVQKLFK   388
KPNIH29__KL64_        PCAINKQLRNFTVVVGEKDSVTGKDIAGLKSELEKQQIKFDYHQYPGLNHEMDVVRPAYAEFVQKLFK   388
ATCC35657__KL64_      PCAINKQLRNFTVVVGEKDSVTGKDIAGLKSELEKQQIKFDYHQYPGLNHEMDVVRPAYAEFVQKLFK   388
NZ_CP098770.1__K47_   PCAINKQLRNFTVVVGEKDSVTGKDIAGLKSELEKQQIKFDYHQYPGLNHEMDVVRPAYAEFVQKLFK   388
```
