# Supplementary material for: A novel esterase regulates Klebsiella pneumoniae hypermucoviscosity and virulence
Source: PLoS Pathog. 2024 Oct 31;20(10):e1012675. doi: 10.1371/journal.ppat.1012675 (PMC11556721; doi:10.1371/journal.ppat.1012675)
Supplement: S5 Fig — (A) Diagram of KpACE (not drawn to scale). (B) Three-dimensional structure of KpACE predicted by AlphaFold. Three putative classical His-Ser-Asp catalytic triads for hydrolysis, including H180-S185-D217, H291-S287-D248, and H370-S278-D339, were predicted in silico and denoted with rectangles. Bidirectional arrows indicate the domains for CBM48 and the Catalytic domain, respectively. (C) Similarity of KpACE (Cyan) and DmCE1B (PDB:7b6b) (Red). (D) The conserved residue H180. (E) The conserved H370-S278-D339 of KpACE (Cyan) with the catalytic triad (H638-S542-E606) of DmCE1B (Red). (F) Multisequence alignment of KpACE and its four homologues. Secondary structural elements of the closest homologue DmCE1B (PDB: 7b6b), a carbohydrate esterase from Dysgonomonas mossii, are shown above the alignment. Additional protein sequences are obtained from acetylesterase/feruloylesterase axe1-6A (AXFA_PRER2) from Prevotella ruminicola, acetyl xylan esterase (Bil1033-CE1) from Bacteroides intestinalis, and feruloyl esterase (Bil1039-CE1) from Bacteroides intestinalis. The catalytic triad (H638-S542-E606) of DmCE1B and conserved residues of Histidine of KpACE are indicated by green and black arrows, respectively. The alignment was created in Clustal Omega and visualized using Espript 3.0. Conserved amino acid residues are displayed as red text on a white background with a blue border. Identical amino acid residues between sequences are displayed in white text on a red background with a blue border. (PDF) [file ppat.1012675.s005.pdf]

S5 Fig. Structural prediction and similarity analysis of KpACE.

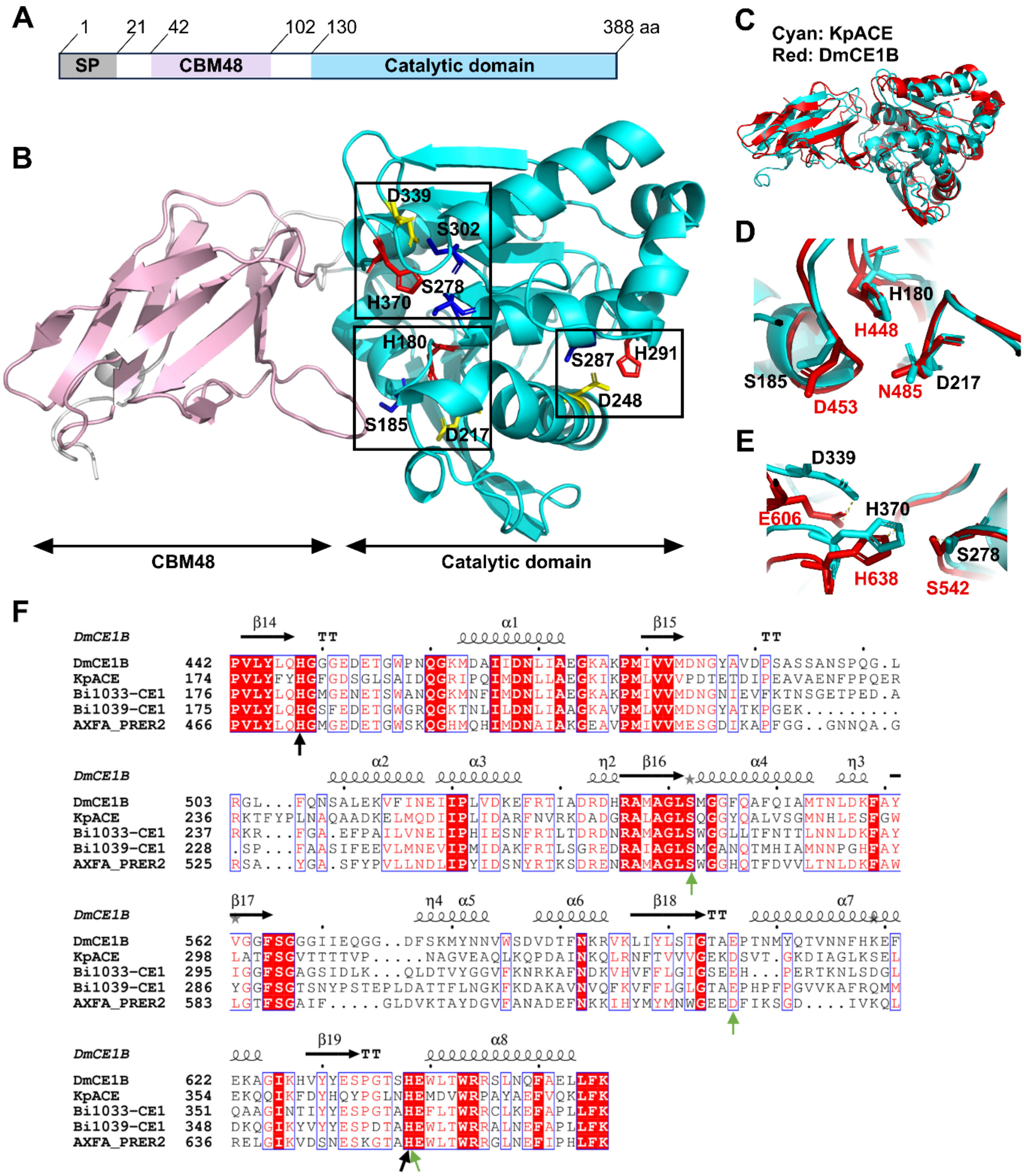

The alignment was created in Clustal Omega and visualized using Espript 3.0 [1, 2].

References:

1. Madeira F, Pearce M, Tivey ARN, Basutkar P, Lee J, Edbali O, et al. Search and sequence analysis tools services from EMBL-EBI in 2022. Nucleic Acids Res. 2022;50(W1):W276-9. Epub 20220412. doi: 10.1093/nar/gkac240. PubMed PMID: 35412617.

2. Robert X, Gouet P. Deciphering key features in protein structures with the new ENDscript server. Nucleic Acids Res. 2014;42(Web Server issue):W320-4. Epub 20140421. doi: 10.1093/nar/gku316. PubMed PMID: 24753421; PubMed Central PMCID: PMCPMC4086106.
