# Supplementary material for: A novel esterase regulates Klebsiella pneumoniae hypermucoviscosity and virulence
Source: PLoS Pathog. 2024 Oct 31;20(10):e1012675. doi: 10.1371/journal.ppat.1012675 (PMC11556721; doi:10.1371/journal.ppat.1012675)
Supplement: S9 Fig — The gene wcsU from 11 clinical strains was amplified and sequenced. Subsequently, the translated protein sequence was aligned with that of ATCC43816. The alignment was created and visualized with DNAMAN 9.0 software. Identical amino acid residues between sequences are displayed in black text on a white background, while variable residues are depicted as black text on a pink or cyan background. The mean identity is 99.59%. (PDF) [file ppat.1012675.s009.pdf]

S9 Fig. Multisequence alignment of WcsU protein from 11 clinical strains of *K. pneumoniae*.

```
ATCC43816   MIFNKVIERISILKNRCRSSECSIIGVLFRMLMFRLVYKKNILTSPKVKIKNIKNIKFHKNSNLIVGLSNVNHVN   75
TH12854     MIFNKVIERISILKNRCRSSECSIIGVLFRMLMFRLVYKKNILTSPKVKIKNIKNIKFHKNSNLIVGLSNVSHVN   75
TH12887     MIFNKVIERISILKNRCRSSECSIIGVLFRMLMFRLVYKKNILTSPKVKIKNIKNIKFHKNSNLIVGLSNVSHVN   75
TH12907     MIFNKVIERISILKNRCRSSECSIIGVLFRMLMFRLVYKKNILTSPKVKIKNIKNIKFHKNSNLIVGLSNVNHVN   75
TH13011     MIFNKVIERISILKNRCRSSECSIIGVLFRMLMFRLVYKKNILTSPKVKIKNIKNIKFHKNSNLIVGLSNVSHVN   75
TH13018     MIFNKVIERISILKNRCRSSECSIIGVLFRMLMFRLVYKKNILTSPKVKIK...NIKFNKNSNLIVGLSNVNHVN   72
TH13021     MIFNKVIERISILKNRCRSSECSIIGVLFRMLMFRLVYKKNILTSPKVKIKNIKNIKFHKNSNLIVGLSNVSHVN   75
TH13022     MIFNKVIERISILKNRCRSSECSIIGVLFRMLMFRLVYKKNILTSPKVKIKNIKNIKFHKNSNLIVGLSNVSHVN   75
TH13026     MIFNKVIERISILKNRCRSSECSIIGVLFRMLMFRLVYKKNILTSPKVKIKNIKNIKFHKNSNLIVGLSNVSHVN   75
TH13030     MIFNKVIERISILKNRCRSSECSIIGVLFRMLMFRLVYKKNILTSPKVKIKNIKNIKFNKNSNLIVGLSNVNHVN   75
TH13034     MIFNKVIERISILKNRCRSSECSIIGVLFRMLMFRLVYKKNILTSPKVKIKNIKNIKFNKNSNLIVGLSNVNHVN   75
TH13035     MIFNKVIERISILKNRCRSSECSIIGVLFRMLMFRLVYKKNILTSPKVKIKNIKNIKFHKNSNLIVGLSNVSHVN   75

ATCC43816   NNSICYINNRGEMHVSGNVFIAKSVRVDIADSSKIILNDCYIGPETDLISYSGISIGKGSMVSVRVQFLDEDFHL   150
TH12854     NNSICYINNRGEMHVSGNVFIAKSVRVDIADSSKIILNDCYIGPETDLISYSGISIGKGSMVSVRVQFLDEDFHL   150
TH12887     NNSICYINNRGEMHVSGNVFIAKSVRVDIADSSKIILNDCYIGPETDLISYSGISIGKGSMVSVRVQFLDEDFHL   150
TH12907     NNSICYINNRGEMHVSGNVFIAKSVRVDIADSSKIILNDCYIGPETDLISYSGISIGKGSMVSVRVQFLDEDFHL   150
TH13011     NNSICYINNRGEMHVSGNVFIAKSVRVDIADSSKIILNDCYIGPETDLISYSGISIGKGSMVSVRVQFLDEDFHL   150
TH13018     NNSICYINNRGEMHVSGNVFIAKSVRVDIADSSKIILNDCYIGPETDLISYSGISIGKGSMVSVRVQFLDEDFHL   147
TH13021     NNSICYINNRGEMHVSGNVFIAKSVRVDIADSSKIILNDCYIGPETDLISYSGISIGKGSMVSVRVQFLDEDFHL   150
TH13022     NNSICYINNRGEMHVSGNVFIAKSVRVDIADSSKIILNDCYIGPETDLISYSGISIGKGSMVSVRVQFLDEDFHL   150
TH13026     NNSICYINNRGEMHVSGNVFIAKSVRVDIADSSKIILNDCYIGPETDLISYSGISIGKGSMVSVRVQFLDEDFHL   150
TH13030     NNSICYINNRGEMHVSGNVFIAKSVRVDIADSSKIILNDCYIGPETDLISYSGISIGKGSMVSVRVQFLDEDFHL   150
TH13034     NNSICYINNRGEMHVSGNVFIAKSVRVDIADSSKIILNDCYIGPETDLISYSGISIGKGSMVSVRVQFLDEDFHL   150
TH13035     NNSICYINNRGEMHVSGNVFIAKSVRVDIADSSKIILNDCYIGPETDLISYSGISIGKGSMVSVRVQFLDEDFHL   150

ATCC43816   VSYNDKKPKDGKITIGENCLIGNNVAINKGCIIADGCVVASHSVVNGVFLEKNCLIAGVPARVIKRNISVQH   222
TH12854     VSYNDKKPKDGKITIGENCLIGNNVAINKGCIIADGCVVASHSVVNGVFLEKNCLIAGVPARVIKRNISVQH   222
TH12887     VSYNDKKPKDGKITIGENCLIGNNVAINKGCIIADGCVVASHSVVNGVFLEKNCLIAGVPARVIKRNISVQH   222
TH12907     VSYNDKKPKDGKITIGENCLIGNNVAINKGCIIADGCVVASHSVVNGVFLEKNCLIAGVPARVIKRNISVQH   222
TH13011     VSYNDKKPKDGKITIGENCLIGNNVAINKGCIIADGCVVASHSVVNGVFLEKNCLIAGVPARVIKRNISVQH   222
TH13018     VSYNDKKPKDGKITIGENCLIGNNVAINKGCIIADGCVVASHSVVNGVFLEKNCLIAGVPARVIKRNISVQH   219
TH13021     VSYNDKKPKDGKITIGENCLIGNNVAINKGCIIADGCVVASHSVVNGVFLEKNCLIAGVPARVIKRNISVQH   222
TH13022     VSYNDKKPKDGKITIGENCLIGNNVAINKGCIIADGCVVASHSVVNGVFLEKNCLIAGVPARVIKRNISVQH   222
TH13026     VSYNDKKPKDGKITIGENCLIGNNVAINKGCIIADGCVVASHSVVNGVFLEKNCLIAGVPARVIKRNISVQH   222
TH13030     VSYNDKKPKDGKITIGENCLIGNNVAINKGCIIADGCVVASHSVVNGVFLEKNCLIAGVPARVIKRNISVQH   222
TH13034     VSYNDKKPKDGKITIGENCLIGNNVAINKGCIIADGCVVASHSVVNGVFLEKNCLIAGVPARVIKRNISVQH   222
TH13035     VSYNDKKPKDGKITIGENCLIGNNVAINKGCIIADGCVVASHSVVNGVFLEKNCLIAGVPARVIKRNISVQH   222
```
