# Supplementary material for: A novel esterase regulates Klebsiella pneumoniae hypermucoviscosity and virulence
Source: PLoS Pathog. 2024 Oct 31;20(10):e1012675. doi: 10.1371/journal.ppat.1012675 (PMC11556721; doi:10.1371/journal.ppat.1012675)
Supplement: S10 Fig — (A) Three-dimensional (3-D) structure of KpACE was predicted using AlphaFold through the Google Colab server. (B) The 3-D structure of one CPS repeat unit was generated by GlyCAM based on the CPS repeat unit of four sugars (upper), and the acetyl group was marked as Ac (bottom) (see Materials and methods). (PDF) [file ppat.1012675.s010.pdf]

S10 Fig. The predicted three-dimensional structures of KpACE and CPS repeat unit.

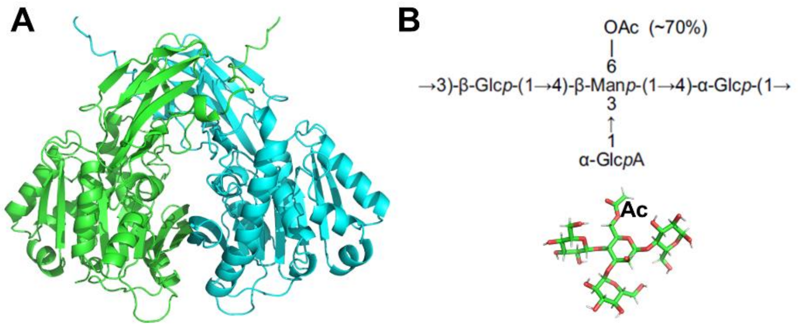

The 3-D structure of one CPS repeat unit was generated by GlyCAM based on the CPS repeat unit of four sugars (upper) [1], and the acetyl group was marked as Ac (bottom) (see Materials and Methods).

Reference:

1. Ovchinnikova OG, Treat LP, Teelucksingh T, Clarke BR, Miner TA, Whitfield C, et al. Hypermucoviscosity regulator RmpD interacts with Wzc and controls capsular polysaccharide chain length. mBio. 2023;14(3):e00800-23. doi: doi:10.1128/mbio.00800-23.
