# Supplementary material for: A novel esterase regulates Klebsiella pneumoniae hypermucoviscosity and virulence
Source: PLoS Pathog. 2024 Oct 31;20(10):e1012675. doi: 10.1371/journal.ppat.1012675 (PMC11556721; doi:10.1371/journal.ppat.1012675)

S11 Fig. *kpACE* overexpression reduces virulence in a pneumonia model using both female and male mice

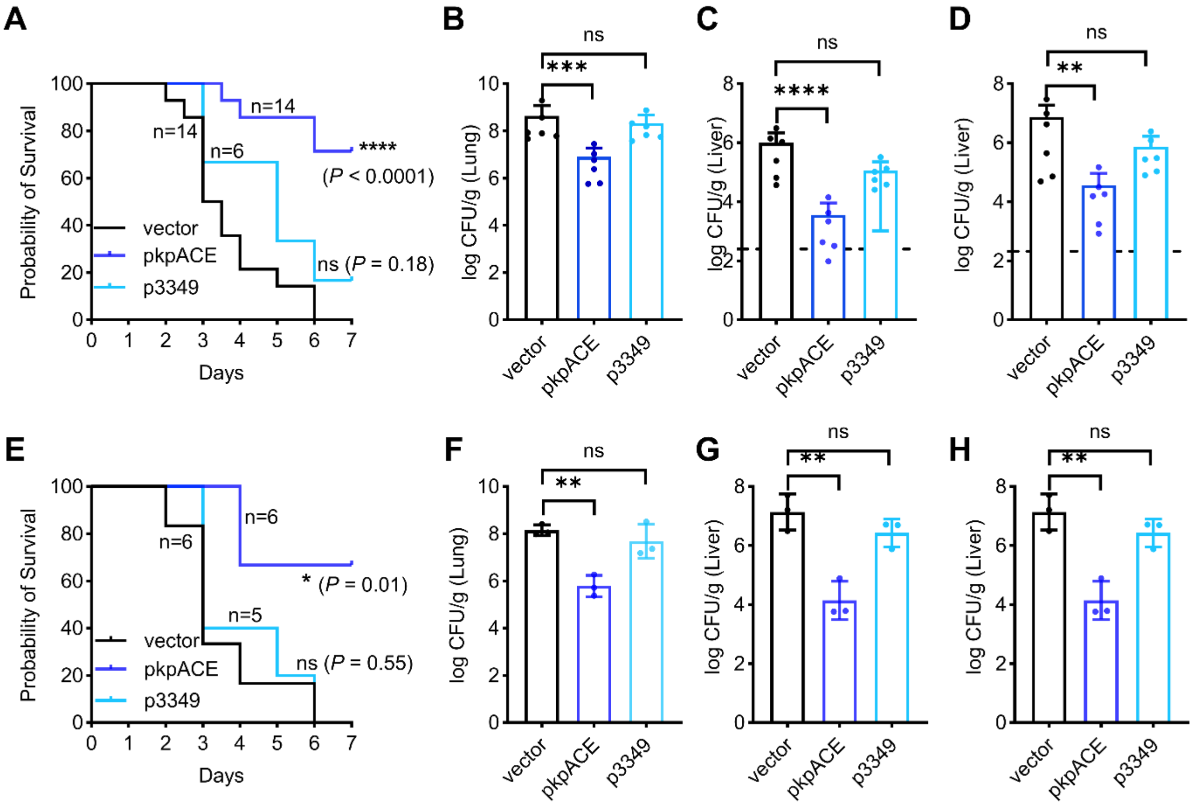

Supplement: S11 Fig — (A-D) kpACE overexpression (pkpACE) reduces virulence in a pneumonia model using female mice. (A) Survival rates of mice. Bacterial burdens in lungs (B), livers (C), and spleens (D) were indicated, respectively. (E-H) kpACE overexpression (pkpACE) reduces virulence in a pneumonia model using male mice. (E) Mouse survival rates of mice. Bacterial burdens in lungs (F), livers (G), and spleens (H) were indicated respectively. Mouse survival rates were monitored 7 days post intranasal infection with 2,000 CFU of ATCC43816 variants. Bacterial burdens in organs were determined 48 h post intranasal inoculation. Each dot represents one mouse, and dotted lines represent the detection limit. The Gehan-Breslow-Wilcoxon test was performed to compare the survival rates. One-way ANOVA with Dunnett’s multiple comparisons test was performed to determine the statistical significance among bacterial burdens in organs. (PDF) [file ppat.1012675.s011.pdf]
