# Supplementary material for: A novel esterase regulates Klebsiella pneumoniae hypermucoviscosity and virulence
Source: PLoS Pathog. 2024 Oct 31;20(10):e1012675. doi: 10.1371/journal.ppat.1012675 (PMC11556721; doi:10.1371/journal.ppat.1012675)
Supplement: S1 Table — (DOCX) [file ppat.1012675.s013.docx]

**S1 Table.** Differentially expressed genes in planktonic cells of the ∆*ompR* mutant

| Gene_ID (VK055_) | Gene | log_2_FC | Product |
| --- | --- | --- | --- |
| 1150 | *pspA* | 1.9 | phage shock protein PspA |
| 1232 | *spy* | 3.8 | ATP-independent periplasmic protein-refolding chaperone |
| 1434 | *putP* | 1.7 | sodium/proline symporter PutP |
| 1436 | *putA* | 2.3 | trifunctional transcriptional regulator |
| 1764 | *galT* | 1.6 | galactose-1-phosphate uridylyltransferase |
| 1765 | *galK* | 1.8 | galactokinase |
| 1766 | *galM* | 1.6 | galactose-1-epimerase |
| 1918 |  | 2.2 | YbdD/YjiX family protein |
| 2532 |  | 2.9 | YgdI/YgdR family lipoprotein |
| 3347 |  | 2.0 | carbohydrate porin |
| 3522 | *lldR* | 2.5 | transcriptional regulator LldR |
| 3523 | *lldP* | 2.4 | L-lactate permease |
| 4934 | *galS* | 2.4 | HTH-type transcriptional regulator GalS |
| 4936 | *mglA* | 3.4 | galactose/methyl galactoside ABC transporter MglA |
| 4937 | *mglC* | 3.1 | galactose/methyl galactoside ABC transporter permease MglC |
| 4943 |  | 2.8 | carbohydrate porin |
| 4948 | *mdtQ* | 1.8 | multidrug resistance outer membrane protein MdtQ |
| 0013 | *ompC* | -3.5 | porin OmpC (OmpK36) |
| 0505 | *dtpA* | -3.3 | dipeptide/tripeptide permease DtpA |
| 0736 |  | -2.2 | DMT family transporter |
| 0739 |  | -2.7 | iron-containing redox enzyme family protein |
| 1527 | *ompF* | -4.0 | porin OmpF (OmpK35) |
| 5052 | *dacD* | -3.7 | serine-type D-Ala-D-Ala carboxypeptidase DacD |
