# Supplementary material for: A novel esterase regulates Klebsiella pneumoniae hypermucoviscosity and virulence
Source: PLoS Pathog. 2024 Oct 31;20(10):e1012675. doi: 10.1371/journal.ppat.1012675 (PMC11556721; doi:10.1371/journal.ppat.1012675)
Supplement: S3 Table — (DOCX) [file ppat.1012675.s015.docx]

S3 Table. Characteristics of strains in this study

| ID | Description | Source |
| --- | --- | --- |
| ATCC43816 | *K. pneumoniae*, serotype K2 | [1] |
| DH5α | *E. coli* DH5α | Lab |
| BL21(DE3) | *E. coli* BL21(DE3) | Lab |
| TH13179 | ATCC43816 pCasKP, Apr^R^ | [1] |
| TH14427 | ATCC43816, ∆*VK055*_3696, ∆*ompR* | [1] |
| TH16429 | ATCC43816, Δ*kpACE* | This study |
| TH16622 | ATCC43816, Δ*wcsU* | This study |
| TH17000 | ATCC43816, Δ*wbbO* | This study |
| TH17001 | ATCC43816, Δ*wbbO* pCasKP, Apr^R^ | This study |
| TH17108 | ATCC43816, Δ*wbbO/kpACE* | This study |
| TH16244 | ATCC43816 pACYC184ΔcatΔtet::PrpsL, Spe^R^ | [1] |
| TH16343 | ATCC43816 p4943, Spe^R^ | This study |
| TH16330 | ATCC43816 p3347 Locus, Spe^R^ | This study |
| TH16355 | ATCC43816 p3347, Spe^R^ | This study |
| TH16356 | ATCC43816 pkpACE, Spe^R^ | This study |
| TH16357 | ATCC43816 p3349, Spe^R^ | This study |
| TH16538 | ATCC43816 pkpACE^CBM^, Spe^R^ | This study |
| TH16539 | ATCC43816 pkpACE^H180A^, Spe^R^ | This study |
| TH16541 | ATCC43816 pkpACE^H370A^, Spe^R^ | This study |
| TH17515 | ATCC43816 pkpACE^Cat^, Spe^R^ | This study |
| TH16447 | *E. coli* BL21 pET28a::*kpACE*, Kan^R^ | This study |
| TH16613 | *E. coli* BL21 pET28a::*kpACE*^H180A^, Kan^R^ | This study |
| TH16614 | *E. coli* BL21 pET28a::*kpACE*^H370A^, Kan^R^ | This study |
| TH12887 | *K. pneumoniae*, clinical strain, serotype K2 | [2] |
| TH12854 | *K. pneumoniae*, clinical strain, serotype K2 | [2] |
| TH12907 | *K. pneumoniae*, clinical strain, serotype K2 | [2] |
| TH13011 | *K. pneumoniae*, clinical strain, serotype K2 | [2] |
| TH13018 | *K. pneumoniae*, clinical strain, serotype K2 | [2] |
| TH13021 | *K. pneumoniae*, clinical strain, serotype K2 | [2] |
| TH13022 | *K. pneumoniae*, clinical strain, serotype K2 | [2] |
| TH13026 | *K. pneumoniae*, clinical strain, serotype K2 | [2] |
| TH13030 | *K. pneumoniae*, clinical strain, serotype K2 | [2] |
| TH13034 | *K. pneumoniae*, clinical strain, serotype K2 | [2] |
| TH13035 | *K. pneumoniae*, clinical strain, serotype K2 | [2] |

1. Wang L, Huang X, Jin Q, Tang J, Zhang H, Zhang JR, et al. Two-component response regulator OmpR regulates mucoviscosity through energy metabolism in *Klebsiella pneumoniae*. Microbiol Spectr. 2023;11(3):e0054423. Epub 20230425. doi: 10.1128/spectrum.00544-23. PubMed PMID: 37097167; PubMed Central PMCID: PMCPMC10269446.

2. Huang X, Li X, An H, Wang J, Ding M, Wang L, et al. Capsule type defines the capability of Klebsiella pneumoniae in evading Kupffer cell capture in the liver. PLoS Pathog. 2022;18(8):e1010693. Epub 20220801. doi: 10.1371/journal.ppat.1010693. PubMed PMID: 35914009; PubMed Central PMCID: PMCPMC9342791.
