# Supplementary material for: A novel esterase regulates Klebsiella pneumoniae hypermucoviscosity and virulence
Source: PLoS Pathog. 2024 Oct 31;20(10):e1012675. doi: 10.1371/journal.ppat.1012675 (PMC11556721; doi:10.1371/journal.ppat.1012675)
Supplement: S4 Table — (DOCX) [file ppat.1012675.s016.docx]

S4 Table. Primers used in this study

| **Primer** | **Sequences(5’-3’)** | **Descriptions** |
| --- | --- | --- |
| Pr18561 | cggcggaattgcagcaacatcaccatcaccatcactagtg | Forward primer to amplify plasmid backbone from pTH16235. |
| Pr18562 | cattggcgtaaataaaacacgacaattatgaggacgcagaattttagg | Reverse primer to amplify plasmid backbone from pTH16235. |
| Pr18559 | cctaaaattctgcgtcctcataattgtcgtgttttatttacgccaatg | Forward primer to amplify *3347 Locus*. |
| Pr18560 | cactagtgatggtgatggtgatgttgctgcaattccgccg | Reverse primer to amplify *3347 Locus*. |
| Pr18557 | ggaaaaccatcgtgaccttccatcaccatcaccatcactagtg | Forward primer to amplify plasmid backbone from pTH16235. |
| Pr18558 | tcaataggcatgcaagtaataatatgtgaggcatttgagaagcacacgg | Reverse primer to amplify plasmid backbone from pTH16235. |
| Pr18555 | ccgtgtgcttctcaaatgcctcacatattattacttgcatgcctattga | Forward primer to amplify *4943*. |
| Pr18556 | cactagtgatggtgatggtgatggaaggtcacgatggttttcc | Reverse primer to amplify *4943*. |
| Pr18577 | gcaggcggaaatctggtggcatcaccatcaccatcactagtg | Forward primer to amplify plasmid backbone from pTH16328. |
| Pr18578 | cactagtgatggtgatggtgatgccaccagatttccgcctgc | Reverse primer to amplify plasmid backbone from pTH16328. |
| Pr18583 | cgagtttgtgcaaaaactgtttaaaCATCACCATCACCATCACtag | Forward primer to amplify plasmid backbone from pTH16235. |
| Pr18584 | cgttcagttatcgtcttaccacattatgaggacgcagaattttagg | Reverse primer to amplify plasmid backbone from pTH16235. |
| Pr18581 | cctaaaattctgcgtcctcataatgtggtaagacgataactgaacg | Forward primer to amplify *kpaxe*. |
| Pr18582 | ctaGTGATGGTGATGGTGATGtttaaacagtttttgcacaaactcg | Reverse primer to amplify *kpaxe*. |
| Pr18579 | cctaaaattctgcgtcctcataattaacctttagggggccttc | Forward primer to amplify plasmid backbone from pTH16328. |
| Pr18580 | gaaggccccctaaaggttaattatgaggacgcagaattttagg | Reverse primer to amplify plasmid backbone from pTH16328. |
| Pr18407 | gaaatttgctaacggcggct | Forward primer to amplify the upstream of *kpACE*. |
| Pr18408 | ctgaaggccccctaaaggttcctctttcgttgtgtccgttc | Reverse primer to amplify the upstream of *kpACE*. |

S4 Table. Primers used in this study (Continued)

| **Primer** | **Sequences(5’-3’)** | **Descriptions** |
| --- | --- | --- |
| Pr18409 | gaacggacacaacgaaagaggaacctttagggggccttcag | Forward primer to amplify the downstream of *kpACE*. |
| Pr18410 | gcaccgctgacaattagcaa | Reverse primer to amplify the downstream of *kpACE*. |
| Pr18569 | tagtgcagctgcgtaactttaccg | Forward sequence to form 20bp spacer sequence before a PAM site to target *kpACE*. The BsaI site is underlined. |
| Pr18570 | aaaccggtaaagttacgcagctgc | Reverse sequence to form 20bp spacer sequence before a PAM site to target *kpACE*. The BsaI site is underlined. |
| Pr18383 | gcgcggcagccatatggcgatgccggacccaag | Forward primer to amplify *kpACE* (26-388 aa). |
| Pr18384 | gctttgttagcagccggatcttatttaaacagtttttgcacaaactcg | Reverse primer to amplify *kpACE* (26-388 aa). |
| Pr18385 | cgagtttgtgcaaaaactgtttaaataagatccggctgctaacaaagc | Forward primer to amplify pET28a. |
| Pr18386 | cttgggtccggcatcgccatatggctgccgcgc | Reverse primer to amplify pET28a. |
| Pr18959 | ggcagtattcttgacgatcggtaagatccggctgctaacaaagc | Forward primer to amplify the plasmid from pTH16447. |
| Pr18960 | gctttgttagcagccggatcttaccgatcgtcaagaatactgcc | Reverse primer to amplify the plasmid from pTH16447. |
| Pr18961 | ggcagtattcttgacgatcggcatcaccatcaccatcactag | Forward primer to amplify the plasmid from pTH16351. |
| Pr18962 | ctagtgatggtgatggtgatgccgatcgtcaagaatactgcc | Reverse primer to amplify the plasmid from pTH16351. |
| Pr18963 | gctgtcgccaaagccagcatagaaatagagcac | Forward primer to amplify the plasmid from pTH16351. |
| Pr18964 | gtgctctatttctatgctggctttggcgacag | Reverse primer to amplify the plasmid from pTH16351. |
| Pr18967 | gtatcccggcctgaacgctgaaatggacgtctg | Forward primer to amplify the plasmid from pTH16351. |
| Pr18968 | cagacgtccatttcagcgttcaggccgggatac | Reverse primer to amplify the plasmid from pTH16351. |
| Pr19000 | gtaatttacgattccaggcaactg | Forward primer to amplify the upstream of *wcsU* (*VK055_5024*). |
| Pr19001 | gataacccttgctggaacgcctacattcagaagatcggcatc | Reverse primer to amplify the upstream of *wcsU* (*VK055_5024*). |
| Pr19002 | gatgccgatcttctgaatgtaggcgttccagcaagggttatc | Forward primer to amplify the downstream of *wcsU* (*VK055_5024*). |

S4 Table. Primers used in this study (Continued)

| **Primer** | **Sequences(5’-3’)** | **Descriptions** |
| --- | --- | --- |
| Pr19003 | aaaccctaaccaaggctgct | Reverse primer to amplify the downstream of *wcsU* (*VK055_5024*). |
| Pr19004 | tagtatatgttatataaataaccg | Forward sequence to form 20bp spacer sequence before a PAM site to target *wcsU* (*VK055_5024*). The BsaI site is underlined. |
| Pr19005 | aaaccggttatttatataacatat | Reverse sequence to form 20bp spacer sequence before a PAM site to target *wcsU* (*VK055_5024*). The BsaI site is underlined. |
| Pr19403 | TAGTAAGCAATTCACCTGAAGAGC | Forward sequence to form 20bp spacer sequence before a PAM site to target *wbbO (VK055_5036)*. The BsaI site is underlined. |
| Pr19404 | AAACGCTCTTCAGGTGAATTGCTT | Reverse sequence to form 20bp spacer sequence before a PAM site to target *wbbO (VK055_5036)*. The BsaI site is underlined. |
| Pr19405 | ACCATCGCCGAAGCATTG | Forward primer to amplify the upstream of *wbbO (VK055_5036)*. |
| Pr19406 | CCGTTTGATCGACAATGCTATGCCCACTTAATGATAC | Reverse primer to amplify the upstream of *wbbO (VK055_5036)*. |
| Pr19407 | ATGAGAAAATTGTGTTTGTCGATCAAACGGTTATCC | Forward primer to amplify the downstream of *wbbO (VK055_5036)*. |
| Pr19408 | GATGAATTCGCGTGAAGCAGT | Reverse primer to amplify the downstream of *wbbO (VK055_5036)*. |
| Pr18319 | ggagggggtgaaagcactc | The forward primer to amplify *rmpD* for qRT-PCR. |
| Pr18320 | atgttctgtgcgagcggaat | The reverse primer to amplify rmpD for qRT-PCR. |
| Pr17406 | cggtctgtcaagtcggatgtg | The forward primer to amplify 16S rRNA for qRT-PCR. |
| Pr17407 | cggaagccacgcctcaag | The reverse primer to amplify 16S rRNA for qRT-PCR. |
| Pr19584 | AAACGGATTGTGACTGCGAC | Forward primer of P3347 (312 bp) |
| Pr19585 | CGCCGAAGCGATCCTGTTTT | Reverse primer of P3347 |
| Pr19576 | CTGGTTTGGTGACAATGCCG | Forward primer for VK055_3347 |
| Pr19577 | GTTTGCCGACCCAGAAATCG | Reverse primer for VK055_3347 |

S4 Table. Primers used in this study (Continued)

| **Primer** | **Sequences(5’-3’)** | **Descriptions** |
| --- | --- | --- |
| Pr19578 | CGCTCGATAGATACCGGCAG | Forward primer for VK055_3348 |
| Pr19579 | GGCGTCCAGACATAGAGACG | Reverse primer for VK055_3348 |
| Pr19580 | CACGCCAAAGCGGATGTATG | Forward primer for VK055_3349 |
| Pr19581 | TCCAGCGTCAGCTTGTGAAT | Reverse primer for VK055_3349 |
| Pr19582 | ATAACCTTGGCGTGGTCTGG | Forward primer for VK055_4943 |
| Pr19583 | CGACGGCGGAGTGGATATAG | Reverse primer for VK055_4943 |
| Pr19927 | GAGCTACAATATGATTTTCAATAAAG | Forward primer for VK055_5024 |
| Pr19928 | AACTCTTAGTGTTGCCATG | Reverse primer for VK055_5024 |
