# Supplementary material for: A novel esterase regulates Klebsiella pneumoniae hypermucoviscosity and virulence
Source: PLoS Pathog. 2024 Oct 31;20(10):e1012675. doi: 10.1371/journal.ppat.1012675 (PMC11556721; doi:10.1371/journal.ppat.1012675)
Supplement: S5 Table — (DOCX) [file ppat.1012675.s017.docx]

S5 Table. Characteristics of isogenic strains constructed in this study

| Strain ID | Genotype | Donor DNA | | Template DNA | Recipient |
| --- | --- | --- | --- | --- | --- |
|  |  | Plasmid | Repair template |  |  |
| TH16323 | pSGKP::kpACE spacer | Pr18569 | Pr18570 | / | DH5α |
| TH16999 | pSGKP::wbbO spacer | Pr19403 | Pr19404 | / | DH5α |
| TH16620 | pSGKP::wcsU spacer | Pr19004 | Pr19005 | / | DH5α |
| TH16429 | *∆kpACE* | pTH16323 | Pr18407/18408  Pr18409/18410 | TH13179 | TH13179 |
| TH16622 | *∆wcsU* | pTH16620 | Pr19000/19001  Pr19002/19003 | TH13179 | TH13179 |
| TH17000 | *∆wbbO* | pTH16999 | Pr19405/19406  Pr19407/19408 | TH13179 | TH13179 |
| TH17108 | *∆kpACE/wbbO* | pTH16323 | Pr18407/18408  Pr18409/18410 | TH13179 | TH17001 |
| TH16328 | p3347 Locus | Pr18561/18562 | Pr18559/18560 | pTH16235/ATCC43816 | DH5α |
| TH16329 | p4943 | Pr18557/18558 | Pr18555/18556 | pTH16235/ATCC43816 | DH5α |
| TH16344 | p3347 | Pr18577 | Pr18578 | pTH16328 | DH5α |
| TH16351 | pkpACE | Pr18583/18584 | Pr18581/18582 | pTH16235/ATCC43816 | DH5α |
| TH16352 | p3349 | Pr18579 | Pr18580 | pTH16328 | DH5α |
| TH16534 | pkpACE^CBM^ | Pr18961 | Pr18962 | pTH16351 | DH5α |
| TH17154 | pkpACE^Cat^ | Pr19502 | Pr19503 | pTH16351 | DH5α |
| TH16535 | pkpACE^H180A^ | Pr18963 | Pr18964 | pTH16351 | DH5α |
| TH16537 | pkpACE^H370A^ | Pr18967 | Pr18968 | pTH16351 | DH5α |
| TH16447 | pET28a::kpACE | Pr18385/18386 | Pr18383/18384 | pET28a/ATCC43816 | BL21(DE3) |

S5 Table. Construction of isogenic strains (Continued)

| Strain ID | Genotype | Donor DNA | | Template DNA | Recipient |
| --- | --- | --- | --- | --- | --- |
|  |  | Plasmid | Repair template |  |  |
| TH16613 | pET28a::kpACE^H180A^ | Pr18385/18386 | Pr18383/18384 | pTH16447/pTH16535 | BL21(DE3) |
| TH16614 | pET28a::kpACE^H370A^ | Pr18385/18386 | Pr18383/18384 | pTH16447/pTH16537 | BL21(DE3) |
