# Supplementary material for: A novel esterase regulates Klebsiella pneumoniae hypermucoviscosity and virulence
Source: PLoS Pathog. 2024 Oct 31;20(10):e1012675. doi: 10.1371/journal.ppat.1012675 (PMC11556721; doi:10.1371/journal.ppat.1012675)
Supplement: Attachment — Submitted filename: 7.12 Response to reviewers.docx [file ppat.1012675.s020.docx]

**Responses to comments**

**Manuscript No**.: PPATHOGENS-D-24-00763

**Title**: A novel esterase regulates *Klebsiella pneumoniae* hypermucoviscosity and virulence

We greatly appreciate the reviewer's constructive comments and suggestions and have revised the manuscript accordingly. Point by point responses were provided below.

**Responses to editor comments:**

1. There are several key issues must be addressed. A major concern raised by both Reviewers 1 and 2 regards the extension of the results to other strains and other capsular serotypes: are these enzymes only important for serotype K2? A phylogenetic analysis would be helpful to put the findings in context.

**Response**: Our data demonstrated the enzymes are important for serotype K2 and the phylogenetic analysis suggest they are also important for other serotypes as well. The manuscript is updated as below.

(Lines 140-146) We analyzed the conservation of KpACE across 1,819 publicly available complete genomes of *K. pneumoniae*. KpACE homologues were identified in 82.9% (1503) of these genomes, with a remarkably high sequence identity of 99.88% (**Supporting Data**). This enzyme is not only prevalent but also frequently found across a variety of K-serotype strains, such as KL64, KL47, KL2, KL1, and others (**S3A Fig** and **S2 Table**). As depicted in **S3B Fig**, the multisequence alignment of KpACE homologues from ten representative strains of *K. pneumoniae* highlights a high degree of conservation, with only a few variations.

(Lines 403-409) Overall, our study demonstrated KpACE is active towards serotype 2 *K. pneumoniae*. However, it is unknow if K2 strain KpACEs are functional in other serotypes of *K. pneumoniae*. The prevalence of KpACE in 82.9% of *K. pneumoniae* genomes and its frequent distribution in various serotypes, KL64, KL47, KL2, and KL1, underscore its importance and the need for further research into its broader biological significance. Future research is required to systematically investigate the role of KpACE in other serotypes of *K. pneumoniae*, utilizing both *in vitro* and *in vivo* models, which would elucidate broader biological significance of KpACE homologues.

2. Also as noted by Reviewers 1 and 2, the strains and constructs should be comprehensively tested in the various described assays.

**Response**: We examined the ∆*wcsU* mutant in the serum killing and biofilm assays as suggested (**Fig 6B and 6C**).

3. Furthermore, as indicated by Reviewer 3, how did you determine the structure of the K2 capsule described in Figure S8 B? What is GlyCAM? If this is predicted, then the algorithm used and the basis for the model should be indicated; if this structure is from a published report (such as Reference 15), then this should be cited. This is important since prior studies suggest that K2 capsule is hyperviscous, but lacks acetylation.

**Response**: The structure of the K2 capsule depicted in S8B Fig is derived from a published report, Reference 15. GlyCAM-Web (www.glycam.org) offers various tools for molecular modeling of oligosaccharides and larger molecular structures containing glycans. We build the 3-D structure of the K2 capsule utilizing the GlyCAM server.

We have updated the figure legend for **S9 Fig** to include a citation to Reference 15 and a description of the methodology in Materials and Methods.

Regarding the statement “K2 capsule is hyperviscous, but lacks acetylation”. Before the publication of the reference 15, no study reported that the K2 capsule is acetylated. We have also shown in this study the K2 capsule is modified with acetylation (Fig. 3C).

4. Lastly, all three reviewers had concerns about missing or inadequate controls that need to be experimentally addressed.

**Response**: We apologize for these oversights and have added controls to ensure the robustness of our findings as suggested: ①A *galF* gene was used as a negative control for qPCR assays in **Fig 1B**. ②The DNA fragment of Pgcv was used as a negative control in **S2 Fig**, which demonstrates specific binding of OmpR to the promoter region of *kpACE* locus. ③An intracellular response regulator VicR was used as a control for subcellular localization in the **S6 Fig**, which demonstrates selective localization of KpACE in supernatants.

5. There are also issues regarding the description of results and conclusions: caution must be used by being specific about the strain or adding appropriate contextualization to help readers understand the extent that the findings are applicable.

**Response**: We greatly appreciate the reviewer's constructive comments and have revised the manuscript accordingly (see below).

(Lines 232-233) The **ATCC43816** CPS biosynthesis locus harbors a gene wcsU (VK055_5024), which encodes a putative acyltransferase.

(Lines 327-328) This dynamic regulation is critical for controlling HMV formation and pathogenesis of **K2 serotype** *K. pneumoniae*.

(Lines 410-411) In summary, this study elucidates the vital function of WcsU and KpACE in regulating the acetylation and deacetylation balance of CPS in **K2 serotype** *K. pneumoniae*.

**Responses to reviewer’s comments:**

**Part I – Summary**

Please use this section to discuss strengths/weaknesses of study, novelty/significance, general execution and scholarship.

**Reviewer #1**: The strength of this study is the topic of hypermucoviscosity in Klebsiella pneumonia and finding genes encoding enzymes that modify the structure. Weaknesses include the lack of clarity was to whether these findings are applicable to strains of other capsular types (are these enzymes only important for serotype K2?) as well as the over interpretation and lack of controls in some of some of the results and experiments.

**Response:** Our study demonstrated that KpACE acts on CPS-K2. Future study is needed to systematically investigate the role of KpACE in other serotype HvKP, utilizing both *in vitro* and *in vivo* models, which should elucidate its broader biological significance.

Controls were now included to ensure the robustness of our findings. ① A *galF* gene was used as a negative control for qPCR assays in **Fig 1B**. ② The DNA fragment of Pgcv was used as a negative control in **S2 Fig**, which demonstrates specific binding of OmpR to the promoter region of *kpACE* locus. ③ An intracellular response regulator VicR was used as a control for subcellular localization in the **S6 Fig**, which demonstrates selective localization of KpACE in supernatants.

**Reviewer #2**: This manuscript reports the findings of a study by Wang, Wang, Zhang, and colleagues regarding the novel role of an acetylesterase and acetyltransferase that acetylate and deacetylate the hypervirulent Klebsiella pneumoniae (Kp) capsule (CPS), the result of which is the modulation of the characteristic hypermucoviscous phenotype. This study builds off of previous work that demonstrated that ompR is required for hypermucoviscosity. Here, the authors show that a small ompR regulon is associated with hypermucoviscosity and narrow this phenotype to a yet uncharacterized gene which they call "KpACE." Overexpression of this gene reduces virulence, and the authors extensively interrogate its potential enzymatic function as an acetylesterase. Correspondingly, they identify an additional acetyltransferase, WcsU, that potentially acetylates CPS, which they also show is necessary for complete virulence. The manuscript is well-written, the data are presented clearly, and I commend the authors for their deep exploration of this interesting phenotype. That said, I feel the authors stopped short of comprehensively demonstrating the association between their genotypes and phenotypes of interest and presenting the strongest possible study.

**Response:** We examined the ∆*wcsU* mutant in the serum killing and biofilm assays as suggested (**Fig 6B and 6C**).

**Reviewer #3**: In the manuscript “A novel esterase regulates Klebsiella pneumoniae hypermucoviscosity and virulence,” Wang et. al have identified and characterized a novel esterase (KpACE) that is regulated by OmpR and influences virulence-associated phenotypes and virulence in a mouse model. They determined that this esterase acts on CPS, removing an acetyl group. When overexpressed, this activity was linked to reduced HMV (an important virulence factor), decreased serum resistance, decreased biofilm production, and reduced virulence in mice. The use of mutants that lost activity in multiple assays strengthen the argument that it is deacetylase activity that led to experimental outcomes. In addition, they provide evidence that a putative acyltransferase encoded in the cps locus appears to be the enzyme that adds the acetyl group to CPS. Strains lacking this gene (wcsU) had similar phenotypes to a strain overexpressing KpACE. Collectively, they conclude that acetylated CPS leads to higher HMV, and thus, increased virulence. As the mechanisms driving HMV are not well characterized, these findings provide insight on a new element contributing to this phenotype that will be beneficial to the Klebsiella field. For the most part the assays are well-controlled, the data are convincing, and the manuscript is well-written.

**Response:** We appreciate this reviewer’s positive comments about thoroughness of our study.

**Part II – Major Issues: Key Experiments Required for Acceptance**

Please use this section to detail the key new experiments or modifications of existing experiments that should be absolutely required to validate study conclusions. Generally, there should be no more than 3 such required experiments or major modifications for a "Major Revision" recommendation. If more than 3 experiments are necessary to validate the study conclusions, then you are encouraged to recommend "Reject".

**Reviewer #1:**

1. The role of at least one of the upregulated genes, 4943, does not appear to be further investigated. Does it make the same amount of capsule (acetylated or not)?

**Response**: Our investigation has been focused on the role of upregulated genes in their potential impact on mucoviscosity. As illustrated in **Fig 1C**, the overexpression of gene 4943 did not result in any significant alteration of this strain's mucoviscosity. Therefore, we did not pursue 4943 further.

2. Line 109: The infections in mice would seem to need much for introduction. In fact, the word “mice” does not even appear in this paragraph.

**Response**: The description has been updated for clarity and providing better introduction as below:

(Lines 117-119) We then evaluated virulence properties of this overexpression strain using a mouse model of pneumonia. The survival rate of mice infected with *K. pneumoniae* overexpressing wild-type KpACE was significantly enhanced (**Fig 1D**).

3. The fact that the overexpression of KpACE results in significantly more capsular polysaccharide (UA in Figure 1H) does not appear to be taken into account in any of the subsequent assays or analyses.

The length of the polysaccharide never seems to be considered.

**Response**: We characterized the strain further using high-performance gel permeation chromatography, a well-established method to determine the size of exopolysaccharides (EPS). No difference was detected. We updated the main text accordingly as described below.

(Lines 350-353) Interestingly, the strain pKpACE overexpressing KpACE, despite having a higher level of CPS, exhibited reduced virulence. This unexpected finding suggests that the modulation of CPS by acetylation, through the action of KpACE, plays a significant role in the virulence of *K. pneumoniae*.

(Lines 130-134) The size of exopolysaccharides (EPS) was also evaluated using the high-performance gel permeation chromatography (HPGPC), a well-established method [1]. The peaks and retention time for the EPS from both strains were similar (**Fig 1I**), indicating that their molecular weights are comparable. These results suggest that the overexpression of KpACE does not alter the length of polysaccharide.

[1].	Goncalves Mdos S, Delattre C, Balestrino D, Charbonnel N, Elboutachfaiti R, Wadouachi A, et al. Anti-biofilm activity: a function of *Klebsiella pneumoniae* capsular polysaccharide. PLoS One. 2014;9(6):e99995. Epub 20140616. doi: 10.1371/journal.pone.0099995. PubMed PMID: 24932475; PubMed Central PMCID: PMCPMC4059750.

4. A double mutant of ∆wbbO and ∆wcsU does not seem to be tested (Figure 3). And a ∆wbbO and pKpACE strain was not tested.

**Response**: We didn’t test the double mutant of ∆*wbbO* and ∆*wcsU* using NMR given the involvement of this approach. To clarify, the absence of the acetyl group in the single ∆*wcsU* mutant implies that this group would also be absent in the double mutant ∆*wbbO*/∆*wcsU*, as depicted in **Fig 3C**.

However, we did measure the levels of acetylation using an established biochemical assay. The significant difference in acetylation was observed between the ∆*wbbO* mutant and the double mutant ∆*wbbO*/*kpACE*, as well as between the wild-type vector control and the pKpACE strain (**Fig 3D and 3E)**. These results suggest that the KpACE influences the acetylation level.

5. The amount of acetylation does not seem to be tested with the KpACE strain (Figure 3C).

**Response**: We did not evaluate the amount of acetylation for the KpACE strain using the NMR as illustrated in Fig. 3C. However, we employed a biochemical assay method to estimate the degree of acetylation. A significant difference in the acetylation amount between the KpACE strain and the corresponding controls were observed and presented in **Figs 3D and 3E**. This data indicates that the KpACE strain has an altered acetylation amount compared to the control strains, which is consistent with the expected enzymatic activity of KpACE on the CPS.

6. The number of mice in these experiments in Figure 6 is not presented.

**Response**: The exact number of mice used in each experimental group was ten, which has been included in the **Fig 6**.

7. It is completely unclear what is being shown in Figure S1. Where are the log2FC values shown?

**Response**: **S1A Fig** is a chord diagram illustrating the top 10 Gene Ontology (GO) terms that are enriched among differentially expressed genes. Additionally, the exact log_2_ fold change (log_2_FC) values for these genes are provided in the **S1 Table**. We updated the figure legend accordingly.

8. Figure S6, why should the protein in the supernatant be larger than that in the pellets.

**Response**: We are also intrigued by this observation and discussed this finding in the discussion session as described below.

(Lines 365-366) Surprisingly, the extracellular form of KpACE is slightly larger than its intracellular precursor. Whether the secreted KpACE is modified and represents a more active form awaits further investigation.

**Reviewer #2**:

1. The authors generated several useful tools (clean KOs of kpACE and wcsU, kpACE, sub-component, and enzymatically dead kpACE overexpression strains) but they are only selectively used. It would greatly enhance the confidence in the authors' conclusions if they used these tools more comprehensively across the study. For example, why did the authors use the kpACE overexpression strain in their lung infection model but not the clean KO? Why did the authors not explore acetyltransferase activity in serum resistance in Figure 5? As a result of their selective use of these strains, the authors fail to fulfill the molecular Koch's postulates, and as such, the strength of the authors' conclusions is limited.

**Response**:

**Concerning the selective use of strains**

We utilized the KpACE overexpression strain in the lung infection model due to its ability to reduce mucoviscosity *in vitro*. Our aim was to assess whether this reduction in mucoviscosity correlates with changes in virulence *in vivo*.

The reason we did not examine the clean KO of *kpACE* in the lung infection model is because the mutant did not alter mucoviscosity *in vitro*.

We agree it is important to explore the role of acetyltransferase activity in serum resistance, and performed the related experiment and presented relevant findings in **Fig 6B**. These results demonstrate that the *wcsU* mutant, deficient in acetyltransferase, exhibited increased sensitivity to serum killing. This finding supports the notion that the acetylation of CPS contributes to the complete virulence of HvKP.

**Concerning the molecular Koch's postulates**

Our primary objective was to elucidate the function of KpACE in altering mucoviscosity in HvKP and concentrated on strains that were most pertinent to this aspect of the research. Utilizing the KpACE overexpression strain in the lung infection model was a strategic choice to assess the impact of increased KpACE levels on HvKP virulence. The results from both the active and enzymatically dead KpACE overexpression strains have provided evidence suggesting a connection between the enzymatic activity of KpACE and the virulence of the bacteria, which aligns with the molecular Koch's postulates.

2. The third catalytic triad (H291) is not described or explored in any detail throughout the study. The data concerning the other triads are convincing, despite being a little confusing regarding the H370 triad-binding phenotype shown in **Fig 4C**, yet this last triad is ignored throughout the manuscript.

**Response**: Our rationale for not studying H291 is based on the findings that only the H370 and H180 triads are conserved in KpACE homologues among the three triads, as depicted in **S4E and S4F** **Fig**, and illustrated in **S4B Fig**. We updated the relevant text as described below.

(Lines 170-172) However, the histidine residue at position 291 is not conserved among KpACE homologues (as shown in **S4F** **Fig**), thus it is not pursued further experimentally.

3. Please explain how KO and other strains were confirmed. Ideally, this would be by whole genome sequence, as the capsule locus is highly susceptible to mutations that can confound experimental phenotypes (see PMID 37610214).

**Response**: The mutants, including TH16429 (Δ*kpACE*), TH16622 (Δ*wcsU*), TH17000 (Δ*wbbo*), and TH17108 (Δ*wbbo/kpACE*), were initially confirmed by PCR of targeted gene loci. We have now performed the whole-genome sequencing as suggested. No additional SNP was found by comparison of their capsule locus. The whole-genome sequencing data and alignment files are attached in the file named '**Supporting Data**'.

**Reviewer #3**: Major Concerns

1. One particular concern stems from the observation that KpACE reduces HMV in several K2 strains. The strain from which the K2 capsule structure was solved lacked detection of an acetyl group (strain Kp52.145, Ref. 24), but is still HMV positive. This point was not addressed and deserves mention in the discussion. Regarding the strains used in Fig. 3B, it would be very useful to know their origin and whether they contain an intact wcsU gene. The loss of HMV following treatment with KpACE cannot fully be attributed to deacetylation because their acetylation status is unknown. I recognize it is unreasonable to perform NMR on all isolates, so extrapolating their acetylation status from presence/absence of wcsU would be very informative and necessary to conclude that the reduction in HMV is due to deacetylation.

That 52.145 is apparently non-acetylated but still HMV, combined with the lack of acetylation information on the K2 collection leaves open the possibility that KpACE has a second function in addition to deacetylation that contributes to reduced HMV. I would not expect a full exploration of this, but the authors should address the caveats of the data.

**Response**:

**Concerning the strain Kp52.145 with HMV but lacing a functional *wcsU*:**

We acknowledge the limitation of our studies and further discussed this point in **Lines 332-342**: However, it has been reported an HMV-positive K2 strain Kp52.145 lacks CPS acetylation due to the absence of an intact wcsU gene. In our study, the Δ*wcsU* mutant of the ATCC43816 strain, also lacks CPS acetylation similar to Kp52.145, but reduces mucoviscosity. It is worth noting that the ATCC43816 Δ*wcsU* did not completely eliminate HMV. This suggests that the CPS acetylation may only partially contribute to the HMV formation, and the cellular context of different strains may mediate HMV differently. These studies also imply that WcsU and KpACE may differentially regulate HMV-independent virulence. In fact, the Δ*wcsU* mutant exhibited enhanced biofilm formation, contrasting with the reduction in biofilm formation observed upon overexpression of KpACE. The KpACE protein sequence of Kp52.145 shares 99.49% identity with that of the ATCC43816 strain, indicating that a functional KpACE is likely expressed in Kp52.145. These studies also highlight that KpACE is multifunctional and may have additional activities beyond deacetylation that contributes to the reduction in HMV formation and virulence.

**Regarding the strains used in Fig. 3B:**

The origin of the strains used in Fig 3B had been listed in S1 Table of our previous publication [2]. As requested, we have now included the citation in the main text (**Line 211**) and in **S3 Table**. In addition, we performed a study that examined wcsU from 11 clinical isolates. The gene *wcsU* from these strains was amplified and sequenced, and the translated protein sequence was aligned with that of ATCC43816. The alignment (**S8 Fig**) demonstrates that these strains contain a functional *wcsU* gene. The presence of *wcsU* implies CPS's acetylation status, which is consistent with the notion that KpACE-mediated deacetylation accounts for the reduction in mucoviscosity following the treatment by KpACE *in vitro*.

[2].	Huang X, Li X, An H, Wang J, Ding M, Wang L, et al. Capsule type defines the capability of Klebsiella pneumoniae in evading Kupffer cell capture in the liver. PLoS Pathog. 2022;18(8):e1010693. Epub 20220801. doi: 10.1371/journal.ppat.1010693. PubMed PMID: 35914009; PubMed Central PMCID: PMCPMC9342791.

2. There are a few conclusions that are overstated. I generally agree with the interpretations, but some wording implies the data are more convincing than they really are.

Some examples:

Line 97: If trying to say that 3347-3349 form an operon, then the wording needs to be softened. Their close proximity on the genome is suggestive on an operon. Use of the word “indicated” led me to think this had been demonstrated experimentally and that was not done.

**Response**: The word “indicated” is changed to “suggested”.

Line 307: Particularly in light of concerns expressed above, in the sentence “This regulatory cycle is pivotal…”, use of pivotal is a bit overstated. Either modify with a qualifier (e.g. data suggest that this cycle is important) or limit this conclusion to strain 43816.

**Response**: We have revised as below:

(Lines 327-328) This dynamic regulation is critical for controlling HMV formation and pathogenesis of **K2 serotype** *K. pneumoniae*.

3. Inclusion of the ∆wcsU mutant in the serum killing and biofilm assays experiments would greatly strengthen their argument for the role of acetylation in these phenotypes.

**Response**: We performed the suggested experiments with the *wcsU* mutant. The results of serum killing and biofilm assays on the ∆*wcsU* mutant were now presented in **Figs 6B** and **6C**. We updated the text as described below.

(Lines 307-309) Effects of the *wcsU* gene on serum killing sensitivity and biofilm formation were investigated, revealing that the Δ*wcsU* mutant exhibited decreased serum resistance compared to the wild-type strain (**Fig 6B**). Conversely, the deletion of the *wcsU* gene enhanced biofilm formation (**Fig 6C**).

4. Two supplemental figures lack important controls

Fig. S2. There is no control for non-specific binding. I sought out Wang, et.al 2023 (Ref. 16) and did not see controls there either, but they did have fragments to which OmpR did not find. However, the units of concentration are different so could not extrapolate to the data herein. Please add some information as to the validity of this being specific binding.

**Response**: We included a control now. The DNA fragment of Pgcv was used as a negative control in **S2 Fig**, demonstrating the specific binding of OmpR to the promoter region of the kpACE locus.

Fig. S6. There are lots of reasons that proteins can show up in multiple fractions that have nothing to do with their localization. Please include control antibodies against a protein that is known to not be secreted. The absence of this protein in the supernatant fraction will be more convincing that KpACE is indeed secreted.

**Response**: We added a control for subcellular localization. An intracellular response regulator VicR was used as a control for subcellular localization in the **S6 Fig**, which highlight selective localization of KpACE in supernatants.

**Part III – Minor Issues: Editorial and Data Presentation Modifications**

Please use this section for editorial suggestions as well as relatively minor modifications of existing data that would enhance clarity.

**Reviewer #1**:

Line 33: This sentence seems like it needs to be switched around and the word “respectively” included.

**Response**: Revised as suggested.

(Lines 34-36) These results highlight the importance of the delicate balance between CPS acetylation by WcsU and deacetylation by KpACE in regulating the pathogenicity of *K. pneumoniae*.

**Reviewer #2**:

1. The authors should include negative controls for qPCR assays (e.g., a gene not in the OmpR regulon).

**Response**: Agreed. A *galF* gene was used as a negative control for qPCR assays in **Fig 1B**.

2. In Figure S2, a negative control (e.g., a gene not in the OmpR regulon) would strengthen the authors' findings.

**Response**: The DNA fragment of Pgcv was used as a negative control in **S2 Fig**, demonstrating the specific binding of OmpR to the promoter region of the kpACE locus.

3. In Figure S3, the authors claim that KpACE is highly conserved yet they only interrogate a handful of strains. Given that there are thousands of publicly available Kp genomes, a more broad interrogation would strengthen the authors' conclusions.

**Response**: We performed the suggested comparison using the publicly available genomes and updated the relevant text as below.

(Lines 140-146) We analyzed the conservation of KpACE across 1,819 publicly available complete genomes of *K. pneumonia*e. KpACE homologues were identified in 82.9% (1503) of these genomes, with a remarkably high sequence identity of 99.88% (Supporting Data). This enzyme is not only prevalent but also frequently found across a variety of K-serotype strains, such as KL64, KL47, KL2, KL1, and others (**S3A Fig and S2 Table**). As depicted in **S3B Fig**, the multisequence alignment of KpACE homologues from ten representative strains of *K. pneumoniae* highlights a high degree of conservation, with only a few variations.

4. The gel in Figure S6 is not high quality. Given that there is a band of roughly equal density connecting lanes 3 and 4, one questions if there was spillover loading the gel that impacts the authors' findings.

**Response**: We repeated the experiment with an intracellular protein as a control for subcellular localization ( **S6 Fig)**.

5. Line 214 should read "harbors."

**Response**: Revised as suggested.

6. Please explain why only one sex was used in the animal studies and if this should be considered a potential confounder.

**Response**: We followed an established model used in previous two publications [3, 4]. Both used female mice for easier handling given there is no gender difference described for *K. pneumoniae* infection and male mice are more aggressive.

[3].	Palacios M, Miner TA, Frederick DR, Sepulveda VE, Quinn JD, Walker KA, et al. Identification of two regulators of virulence that are conserved in *Klebsiella pneumoniae* classical and hypervirulent strains. mBio. 2018;9(4):e01443-18. Epub 2018/08/09. doi: 10.1128/mBio.01443-18. PubMed PMID: 30087173; PubMed Central PMCID: PMCPMC6083908.

[4].	Lawlor MS, Hsu J, Rick PD, Miller VL. Identification of *Klebsiella pneumoniae* virulence determinants using an intranasal infection model. Mol Microbiol. 2005;58(4):1054-73. doi: 10.1111/j.1365-2958.2005.04918.x. PubMed PMID: 16262790.

7. Regarding the discrepancy in the acetylation of CPS in citations 26 and 27, what do the authors think is the cause of the different observations?

**Response**: Many factors may be accounted for the discrepancy in the acetylation of CPS as observed in two references. One possible reason is the sensitivity of *O*-acetyl group to pH levels. Variations in the pH during the purification process of CPS could lead to differences in the degree of acetyl group removed during the process. Additionally, the techniques used for purification and the specific conditions under which NMR spectroscopy is performed might also contribute to the observed differences in acetylation. It's important to consider the methodology and conditions under which the samples were prepared and analyzed to fully understand the reasons underlying such discrepancies.

8. Do the authors think that their phenotype is limited to K2 strains?

**Response**: Based on the current study, we conclude that the enzyme KpACE is selective for K2-CPS and its activity on this substrate is related to the *O*-acetylation status of the CPS. However, the distribution of KpACE in other serotypes and its high conservation imply the potential function of KpACE in the other serotype strains (**S3A Fig and S2 Table**). Future research is needed to systematically investigate the role of KpACE in other serotype HvKP, utilizing both *in vitro* and *in vivo* models, which would elucidate its broader biological significance. We updated our discussion accordingly.

**Reviewer #3**: Minor Concerns

1. Although not essential, it would be very interesting to know if KpACE could reduce HMV from other K types such as K1 or others that are associated with HMV.

**Response**: We don’t have data that address the effects of KpACE on HMV of other K-serotype strains. However, KpACE is prevalent across a variety of K-serotype strains (**S3A Fig and S2 Table**), suggesting its function may be conserved as well. Further research would be necessary to investigate this possibility, including *in vitro* and *in vivo* experiments to assess the activity and specificity of KpACE against different K serotypes.

2. I did not see any information in Material & Methods describing how the cultures were grown with recombinant KpACE (Fig. 3A).

**Response**: The cultures were grown in LB broth supplemented with different concentrations of the recombinant KpACE protein. The information is now included in Material & Methods and the figure legend of Fig 3A.

3. The sentence that begins on line 266 “The loss of activity…” is very confusing.

**Response**: We revised the sentence for clarity and accuracy as described below:

(Lines 284-287) The pKpACE^H370A^ mutant abrogated its catalytic activity, and reversed the increased susceptibility to serum killing observed in the pKpACE overexpressing strain. This suggests that the esterase activity of KpACE is crucial and likely modulates its interaction with the CPS, thereby affecting serum killing sensitivity.

4. Line 154, a sentence suggests that a mutation (His 180), rather than the mutant protein, has retained its activity.

**Response**: The sentence in question indeed refers to the His180 residue within the protein sequence. We have revised the sentence at Line **169**.

5. Line 109, this sentence reads as if the bacteria had higher survival rates, rather than the mice.

**Response**: Revised as below.

(Lines 117-119) We then evaluated virulence properties of this overexpression strain using a mouse model of pneumonia. The survival rate of mice infected with *K. pneumoniae* overexpressing wild-type KpACE was significantly enhanced (**Fig 1D**).

6. Please consider larger fonts on all your graphs. They are very hard to read!

**Response**: We apologize for not using larger fonts and increase the font size to 12 now (Figs 1-6).

7. Is the RNA-seq data in Table S1 separate from that in Ref. 16? If so, please expand the first paragraph of the results to more comprehensively explain this, and provide information on how to access the raw data (if required by journal). If it’s the same data set, that needs to be indicated.

**Response**: Yes, the RNA-seq data presented in **S1 Table** is new, not from the data set referenced in Ref.16. We have expanded the first paragraph of the results section to clearly state that the data in S1 Table represents a new RNA-seq experiment as described below.

(Lines 92-95) To comprehensively determine the molecular mechanisms involved in HMV formation, we performed an additional RNA-seq analysis of *K. pneumoniae* grown at log-phase to identify the OmpR regulon associated with HMV.

Line 995: Data and materials availability: Raw RNA-seq data are available in NCBI's GEO database (accession number GSE205098).
