# Supplementary material for: A novel esterase regulates Klebsiella pneumoniae hypermucoviscosity and virulence
Source: PLoS Pathog. 2024 Oct 31;20(10):e1012675. doi: 10.1371/journal.ppat.1012675 (PMC11556721; doi:10.1371/journal.ppat.1012675)
Supplement: Attachment — Submitted filename: response letter-09-22.docx [file ppat.1012675.s021.docx]

**Responses to reviewer comments**

**Manuscript No**.: PPATHOGENS-D-24-00763

**Title**: A novel esterase regulates *Klebsiella pneumoniae* hypermucoviscosity and virulence

We greatly appreciate the reviewer's constructive comments and suggestions and have revised the manuscript accordingly. Point by point responses were provided below.

**Part I - Summary**

Reviewer #1: The authors have adequately addressed my previous concerns.

**Response**: Thanks

Reviewer #2: This manuscript is a resubmission of a study by Wang, Wang, and Zhang. The authors have addressed several, but not all of my concerns. Th inclusion of data supporting a more universal prevalence of KpACE enhances the impact of this study, and the additional controls improves the confidence of the authors findings. Yet, the authors' have yet to fulfill the molecular postulates, and potential sex biases of their findings quench the excitement of the new data they have provided.

**Response:** We performed additional animal experiments with male mice, which showed the same outcome trends as the female mice. Figures 1D-G and 6D-G have been revised to include these new results.

We believe our studies demonstrated the importance of acetylation in regulating HMV and virulence as we determined that both the reduced acetylation by the overexpression of KpACE and the elimination of acetylation by the WcsU mutation reduced HMV and diminished virulence, which fulfills the molecular postulate.

At the request of this reviewer, we performed the study by infecting mice using the KpACE knockout mutant. Much to our surprise, the knockout did not alter bacterial virulence, suggesting the regulation of acetylation by KpACE is complex. It is possible that other unknown KpACE-like enzymes exist in this bacterium that can compensate for the lack of KpACE. Another possibility is that the animal model used in the virulence study did not have the needed resolution to resolve the subtle difference between the wild type *K. pneumoniae* and its *KpACE* mutant. This study also suggests the finetuned regulation of KpACE is essential for its manifestation in reducing HMV and virulence. We discussed these possibilities in the discussion section now. Nevertheless, this new result did not diminish the importance of our study in elucidating the dedicate balance between acetylation and deacetylation in regulating HMV and virulence. Our studies further suggest that the protective effect of KpACE is dependent on its elevated expression levels. Our study also highlights the therapeutic potential of this enzyme in treating Klebsiella infections. This is new and significant, and of course warrants additional research given its complexity.

Reviewer #3: In this revised manuscript (PPATHOGENS-D-24-00763-R1), the authors appear to have addressed comments satisfactorily. It is a much smoother read and the additional data and controls have strengthened the conclusions. I have only a few minor comments for consideration.

**Response:** As suggested, additional explanation for the RNA-seq experiment of log-phase bacterial cells has been added in Materials and Methods.

**Part II – Major Issues: Key Experiments Required for Acceptance**

Reviewer #1: (No Response)

Reviewer #2:

1. Regarding the use of the KpACE overexpression strain, I understand the reasons for using this strain in general; however, the role of mucoviscosity in lung infection is well-established. Thus, the authors are not substantially advancing our understanding of Kp infection, as this strain is experimentally useful but biologically artificial. In the absence of the clean KO, the authors can only conclude that artificial repression of mucoviscosity via KpACE reduces infectivity, rather than KpACE being a virulence factor. The inclusion of strains of interest in the in vitro approaches strengthens the authors conclusions, but the definition of KpACE as a virulence factor seems to be a keystone finding in this study. Without such evidence, the novelty of the authors findings are diminished.

**Responses:** We performed suggested studies with a clean KpACE knockout mutant. Interestingly the mutant did not exhibit enhanced virulence. Please see our responses in the Part I.

In addition, the impact of elevated KpACE on virulence was not artificial, we discovered this through the study of a HMV mutant, an *ompR* deficient strain. This defective strain exhibited elevated expression of KpACE, reduced HMV and decreased virulence. This manuscript highlights the importance of our study in elucidating the dedicate balance between acetylation and deacetylation in regulating HMV and virulence, a topic has not been studied before.

2. Regarding sex as a biological variable, I am not convinced of the authors' arguments. The majority of infection models now use both sexes to ensure there is not a sex bias in the study findings (see PMC8221458 for a topical review). I appreciate that the referenced studies only included female mice, but that is not sufficient justification to ignore sex as a potential confounder in the present study.

**Response:** We have conducted additional animal experiments with male mice, which showed the same outcome trends as the female mice. Figures 1D-G and 6D-G have been revised to include these new findings.

Reviewer #3: None

**Part III – Minor Issues: Editorial and Data Presentation Modifications**

Reviewer #1: (No Response)

Reviewer #2: None

Reviewer #3:

1. Some additional explanation for the new RNA-seq experiment would help distinguish this study from the previous one (Ref. 16). Wang, et al. 2023 also states that the RNA-seq experiment was to determine the” OmpR regulon involved in mucoviscosity”. Looking deeper into the Materials & Methods, it appears different media and growth conditions were used—but why?? I realize few readers will notice or care, but as a fan of both HMV and transcriptional regulation, I dig deep into these details. (Also, in Wang, et al. 2023, media listed as “BAP” but this was never defined—assuming Blood Agar Plates?)

**Response:** In Materials and Methods, additional explanation for the RNA-seq experiment of log-phase bacterial cells has been added.

**Lines 443-444:** Distinct genes may be involved in the formation of HMV phenotype in cells at the log-phase and stationary phases, as documented by previous study on the OmpR function (Bang et al., 2000).

The rationale of RNA-seq experiment:

The two-component response regulator governs transcriptional regulation, a process frequently influenced by the growth phase (Bergkessel, 2021). Distinct genes may be involved in the formation of HMV phenotype in cells at the log-phase and stationary phases, as suggested by previous research on the function of the OmpR (Bang et al., 2000). Consequently, our transcriptomic studies encompassed cells from two distinct growth phases: logarithmic and stationary. This strategy enables us to systematically map HMV-related targets of OmpR.

Yes, BAP media refers to Blood Agar Plate, a medium commonly utilized in clinical settings to assess the HMV phenotype of hv-KP. The appearance of hv-KP colonies in a stationary growth phase on BAP can serve as an indicator of their HMV phenotype. Consequently, transcriptomic analysis of these colony cells on BAP could accurately identify the stationary-phase targets regulated by the OmpR.

**References:**

Bergkessel, M. (2021). Bacterial transcription during growth arrest. Transcription *12*, 232-249.

Bang, I.S., Kim, B.H., Foster, J.W., and Park, Y.K. (2000). OmpR regulates the stationary-phase acid tolerance response of *Salmonella enterica* serovar Typhimurium. J Bacteriol *182*, 2245-2252.

2. Line 157, did the authors perhaps mean “potential” instead of “potent”?

**Response:** Yes, it is revised as potential.

3. Lines 286-287, in the last sentence, the highlighting that the interaction between CPS & KpACE affected serum killing is a little bit illogical. Not untrue, but it confused me a bit.

**Response:** This sentence is revised as below.

**Lines 290-291:** This suggests that the esterase activity of the KpACE protein plays a crucial role and exerts its effect on CPS, which influences the bacteria's sensitivity to serum killing.

4. I appreciate the added phylogenetic analysis of KpACE genes, but the high sequence similarity made me wonder if the purified enzyme from this K2 strain could be used to treat other HMV+ Klebsiella strains? Having better addressed in this revised version that the broader application of this data to other K types can only be speculated means this experiment is not essential. However, if expression and purification of this enzyme are not exceedingly laborious, this information would greatly enhance the impact of this study on the field of Klebsiella virulence.

**Response:** We agree that additional studies of protection against other *K. pneumoniae* serotypes by this KpACE would enhance the impact of our study. The current study focuses on understanding the underlying mechanisms, and we will pursue this line of investigation as we are very interested in exploring the therapeutic potential of KpACE in combating infections by various serotypes of hvKP.
