# Supplementary material for: A novel esterase regulates Klebsiella pneumoniae hypermucoviscosity and virulence
Source: PLoS Pathog. 2024 Oct 31;20(10):e1012675. doi: 10.1371/journal.ppat.1012675 (PMC11556721; doi:10.1371/journal.ppat.1012675)
Supplement: Attachment — Submitted filename: response letter 10.2.docx [file ppat.1012675.s022.docx]

**Responses to comments**

**Manuscript No**.: PPATHOGENS-D-24-00763

**Title**: A novel esterase regulates *Klebsiella pneumoniae* hypermucoviscosity and virulence

We greatly appreciate the constructive comments and suggestions and have revised the manuscript accordingly. Point by point responses were provided below.

1. Infection with male and female mice is not indicated in the legend. This should be added to figure 1 and 6.

Response: Infection with male and female mice is now indicated in both figure legends.

2. The results for male and female animals is not shown. This should be added as a supplemental figure.

Response: The separate results for male and female animals are now added as Figs S11 and S12. The combined data are presented in the Figure 1 and 6, and infection with both male and female mice now indicated in their legends, respectively.

3. S3 Fig. Effects of KpACE deficiency on virulence. The sex of the mice is not indicated.

Response: The sex of mice is now included in S3 Fig legend as below.

(A) Survival rates of mice (six female and four male) infected by *K. pneumoniae* variants. (B-D) Bacterial colonization in different organs of infected mice (three female and three male).
